# Spatial prediction of the probability of liver fluke infection in water resource within sub-basin using an optimized geographically-weighted regression model

**DOI:** 10.3389/fvets.2024.1487222

**Published:** 2024-11-07

**Authors:** Benjamabhorn Pumhirunroj, Patiwat Littidej, Thidarut Boonmars, Atchara Artchayasawat, Nutchanat Buasri, Donald Slack

**Affiliations:** ^1^Program in Animal Science, Faculty of Agricultural Technology, Sakon Nakhon Rajabhat University, Sakon Nakhon, Thailand; ^2^Research Unit of Geoinformatics for Spatial Management, Department of Geoinformatics, Faculty of Informatics, Mahasarakham University, Maha Sarakham, Thailand; ^3^Department of Parasitology, Faculty of Medicine, Khon Kaen University, Khon Kaen, Thailand; ^4^Department of Agriculture and Resources, Faculty of Natural Resources and Agro-Industry, Kasetsart University, Chalermphrakiat Sakon Nakhon Province Campus, Sakon Nakhon, Thailand; ^5^Department of Civil and Architectural Engineering and Mechanics, University of Arizona, Tucson, AZ, United States

**Keywords:** liver fluke, Opisthorchis viverrini (OV), spatial modeling, geographically-weighted regression (GWR), Sakon Nakhon, Thailand, connection Mekong River

## Abstract

**Introduction:**

Infection with liver flukes (Opisthorchis viverrini) is partly attributed to their ability to thrive in sub-basin habitats, causing the intermediate host to remain within the watershed system throughout the year. It is crucial to conduct spatial monitoring of fluke infection at a small basin analysis scale as it helps in studying the spatial factors influencing these infections. The number of infected individuals was obtained from local authorities, converted into a percentage, and visually represented as raster data through a heat map. This approach generates continuous data with dependent variables.

**Methods:**

The independent set comprises nine variables, including both vector and raster data, that establish a connection between the location of an infected person and their village. Design spatial units optimized for geo-weighted modeling by utilizing a clustering and overlay approach, thereby facilitating the optimal prediction of alternative models for infection.

**Results and discussion:**

The Model-3 demonstrated the strongest correlation between the variables X5 (stream) and X7 (ndmi), which are associated with the percentage of infected individuals. The statistical analysis showed t-statistics values of −2.045 and 0.784, with corresponding *p*-values of 0.016 and 0.085. The RMSE was determined to be 2.571%, and the AUC was 0.659, providing support for these findings. Several alternative models were tested, and a generalized mathematical model was developed to incorporate the independent variables. This new model improved the accuracy of the GWR model by 5.75% and increased the *R*^2^ value from 0.754 to 0.800. Additionally, spatial autocorrelation confirmed the difference in predictions between the modeled and actual infection values. This study demonstrates that when using GWR to create spatial models at the sub-basin level, it is possible to identify variables that are associated with liver fluke infection.

## 1 Introduction

OV infection is endemic in Southeast Asia, particularly along the Mekong Basin, which includes Thailand, Lao PDR, and Cambodia (1–4), where it is estimated that nine million individuals are infected. Transmission to humans and other animals occurs through the consumption of uncooked cyprinoid or white-scale freshwater fish containing the infective stage metacercariae. Following infection, OV persists in the bile duct in the absence of treatment. Numerous studies have established a strong correlation between chronic infection and bile duct cancer, specifically cholangiocarcinoma (CCA) (5–8). The International Agency for Research on Cancer has classified OV as a group 1 agent, carcinogenic to humans. Thailand reports the highest CCA incidence globally, with estimates ranging from 93.8 to 317.6 cases per 100,000 person-years (5, 9–11). Nevertheless, OV infection is recognized as a neglected and underestimated disease on a global scale. Severe liver fluke infections have been discovered in Ponna Kaeo district, Sakon Nakhon province, Thailand. The liver fluke, Opisthorchis viverrini, is responsible for causing cholangiocarcinoma (CCA) (12–14). Thailand has the highest prevalence of bile duct cancer cases due to liver fluke infection (5). This infection occurs when raw fish, contaminated with infectious larvae, is consumed, along with the widespread consumption of semi-raw or raw seafood. Fluke infections have also been reported from fermented fish products (15). Each year, Sakon Nakhon Hospital identifies nearly a thousand new cases of CCA. Despite knowing the primary risk factors for O. viverrini infection, the incidence of CCA has not decreased in the past decade (16, 17). The prevalence of CCA in Thailand's four main regions—Sakon Nakhon, Phrae, Roi-Et, and Nong Bua Lamphu—remains unknown (12, 18, 19). Individuals with a high severity of O. viverrini infection (>6,000 eggs/g. feces) have a 14.1-fold increased probability of developing CCA compared to those without the infection (20). Approximately 10% of people with O. viverrini infection progress to CCA, leading to significant health crises in the region (21, 22). The 5-year survival rates for patients with intrahepatic, distal extrahepatic, and hilar CCA who undergo surgery are 22–44, 27–37, and 11–41% respectively, according to Hasegawa et al. (23).

The largest natural water contact zone in the northeast is located near the boundary of the Nong Han subdistrict due to the unique topography of the area. The physical characteristics of the swamp make it a significant natural water source that remains full at all times. This is because it is fed by multiple streams along the shoreline, making it an essential source of food for the locals. Fish, which is a major protein source for people in the watershed, is consumed raw or cooked with herbs, giving it a delightful sweet, sour, and spicy flavor (14, 24). As a result, residents living near the river basin typically include fish in every meal. Preliminary screening results from 2019 to 2021 indicate that only a small percentage of people have contracted liver fluke (12). Moreover, research on fish liver fluke infection prevalence (contagious larvae) has shown that the Sakon Nakhon province has an infection rate of 33.33% (21). A study conducted in 2016–2017 on the density of contact larvae in fish found that there were 10–20 metacercaria per kilogram of fish (20). As a result, Sakon Nakhon province continues to experience outbreaks of liver fluke, as the feces containing the parasite's eggs may contaminate water sources and lead to recurring illnesses and an ongoing cycle of infection.

The use of geographically information system (GIS) knowledge as an analytical tool is particularly valuable in studying liver fluke infections through remote sensing information systems. Remote sensing (RS) derived from satellite imagery allows for in-depth analysis of the likelihood and distribution of liver flukes. This analysis can involve various indicators, such as the standardized vegetation index, soil moisture index, soil cover index, and other indices that may be associated with the presence of liver fluke intermediates (22, 25).

Many studies have used spatial statistics to investigate the correlation between geographicallyal factors and liver fluke infection (26). However, there have been conflicting results and inconsistencies in the raster data due to the analysis of large areas in some studies (22, 27). In contrast, other studies (28–30), have focused on creating geographically-weighted regression (GWR) models in smaller area units for hydrological factor analysis, which have yielded high *R*^2^ values in all models. Combining proper spatial modeling with mathematical models can improve the accuracy of linear models, as demonstrated in a study by Sangpradid (31). There is also a study of Littidej et al. (32) in concerning the creating one of the independent variable in areas where the density of the dependent variable is similar comparable.

However, the principles of geo-statistics (33), and, specifically, the GWR modeling method require the creation of sub-spatial units (28), such as sub-basins. These sub-basins are defined from the flow boundary of the sub-basin to the modeling control boundary, and they are necessary in accurately analyzing the numerous indices that must be constructed as independent variables. As stated by Lu et al. (34), this makes GWR models effective in forecasting and understanding spatial correlations. To create spatial models for studying relationships in small areas, like sub-basin levels (35), it is essential to use the appropriate models and tailor the sub-area units to the distribution of data and dependent and independent variables. Relying solely on OLS models in independent multivariate analysis often leads to low accuracy due to the many independent elements that contribute to the model's variability. However, in this study, GWR modeling was used to examine the association between a collection of independent variables and the proportion of infections prior to OV. Previous spatial modeling research did not incorporate GWR models or sub-spatial unit boundaries in small watershed systems to detect liver fluke infections. This study aims to identify the independent variables involved in spatial infections and accurately model them using a limited collection of connected independent variables through GWR modeling.

Therefore, effective management of the sub-basin level can ensure protection, as long as it can be demonstrated that the spatial distribution of each parasite's features is significant within each sub-basin unit (36). For example, by disrupting the mollusc host cycle, we can promote the wellbeing of populations and prevent future diseases, resulting in reduced community impact and medical expenses.

Based on previous research, this study discovered the following guidelines for utilizing the model: A spatial model was created to examine the spatial characteristics of liver fluke infection, with two main objectives: 1. analyzing the spatial factors linked to human liver fluke infection based on sub-basin boundaries, and 2. developing an alternative model to enhance the effectiveness of preventive public health management in order to lower the risk of liver fluke infection in humans.

## 2 Materials and methods

### 2.1 The study area

**Figure 1:**
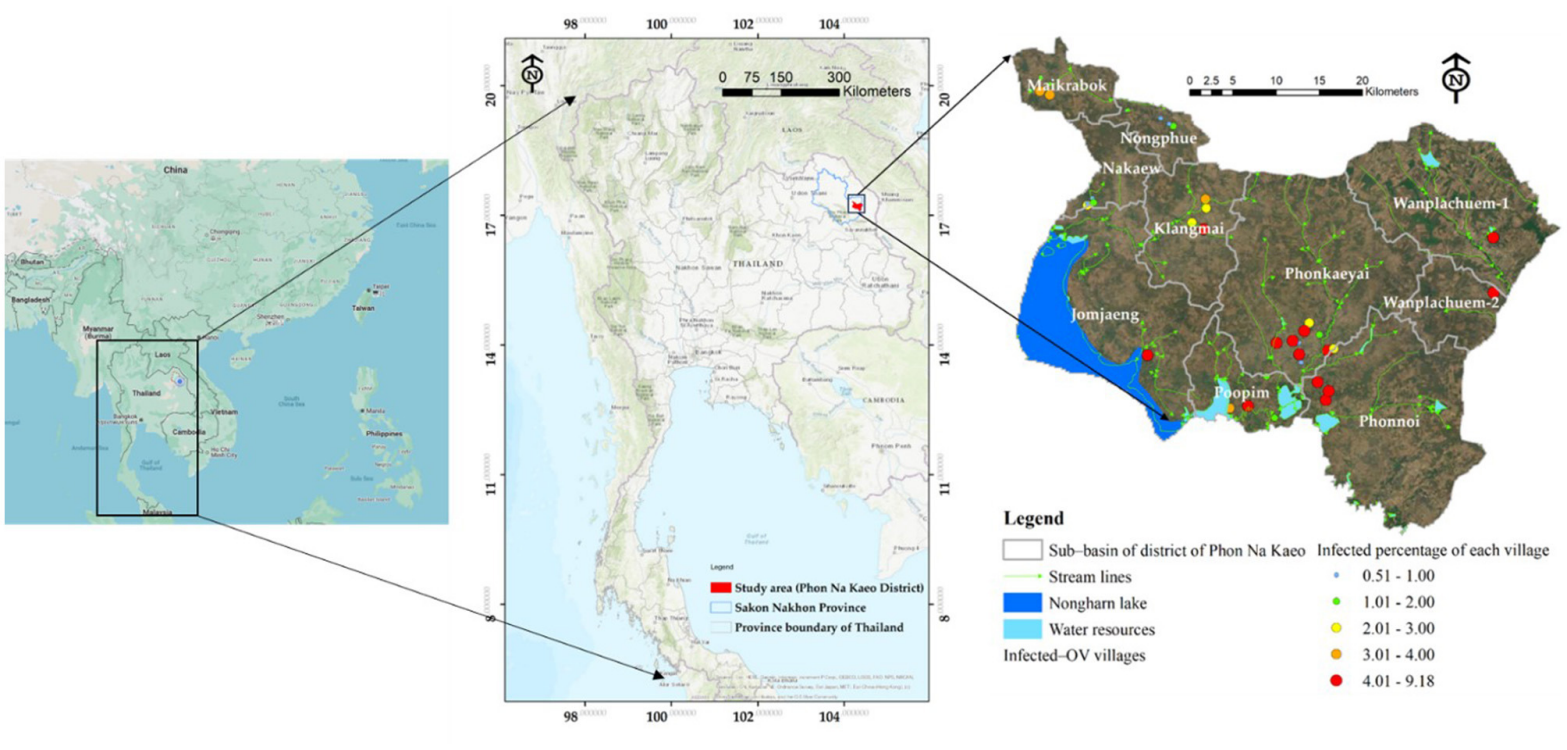
The study area and distribution of prevalence of ov-infection in term of percentage.

This study developed a prototype model in the upper northeastern region of Thailand, specifically in Sakon Nakhon Province. The model concentrated on the district where the river outlet connects to the Mekong River. Phon Na Kaeo district in Sakon Nakhon province shares its borders with Kusumal district to the north. To the east, it is bordered by Pla Pak district and Wangyang district, while to the south it borders Khok Si Suphan district, Mueang Sakon Nakhon district, and Wangyang district. On the west side, it shares its border with Mueang Sakon Nakhon district. The district's geographically coordinates, as shown in Figure 1, are 17 °13′18^′′^N, 104°17′24^′′^E. There are five sub-districts in Sakon Nakhon province's Phon Na Kaeo district: Ban Phon, Na Kaeo, Nadong Wattana, Ban Khae, and Chiang Shi. This district is situated to the east of the Songkram watershed and is in close proximity to Nakhon Phanom province and the Nong Harn marsh, which is a large natural water source. Due to its proximity to the Mekong River, which is around 40 km away, there is an exchange of Mekong fish and fish habitat in the Phon Na Kaeo district. This can result in the movement of numerous Mekong/tributary fish in the area, as well as potentially increasing the number of liver fluke infections in fish.

### 2.2 Analyses and datasets

In Thailand, there are significant public health concerns that have persisted for a long time. These include liver fluke and cholangiocarcinoma, a deadly disease that claims the lives of at least 20,000 people each year in the northeast region (37, 38). With a current estimate of 6–8 million cases of liver fluke infection, it is absolutely crucial to test individuals for this infection. By eliminating the parasites, we can reduce the risk of developing cholangiocarcinoma (39). The Sakon Nakhon Provincial Public Health Office (SKKO) (40) provided the information on liver fluke infections used in this study. A common screening technique that has been utilized for many years is stool testing. For instance, the modified Kato-Katz approach, which has proven to be an efficient way in the past when there were widespread parasite outbreaks, can be used to examine parasite eggs in feces in-depth. In addition, stool analysis has been a well-established procedure for many years. Using the modified Kato-Katz technique, stool samples were examined for O. viverrini eggs shortly after collection (41). The majority of infected individuals were found in the Phon Na Kaeo district of Sakon Nakhon province. The infection prevalence tends to increase among individuals aged 18–80 years. Two other testing techniques, namely the enzyme-linked immunosorbent assay (ELISA) and the formalin-ethyl acetate concentration technique (FECT), are more effective than stool samples (42). Additionally, they provide numerical data that can be correlated with parasite density and used for post-drug evaluations to determine the rate of new or reinfected infections (36, 41, 42). However, these approaches would have required a substantial budget, which is why they were not utilized in this study. Nevertheless, the modified Kato-Katz method is a suitable technique for assessing a large number of individuals, and the secondary data obtained from SKKO regarding the number of liver fluke-infected patients assessed using this approach is reliable. According to data on modified Kato-Katz fluke infections, the highest number of cases was reported in the Phon Na Kaeo district of Sakon Nakhon province. The prevalence of infection tended to increase in the age group of 30–40 years. In terms of patient infection density, it was found to be similar to the prevalence, indicating that the province had the highest concentration of liver fluke infections.

**Figure 2:**
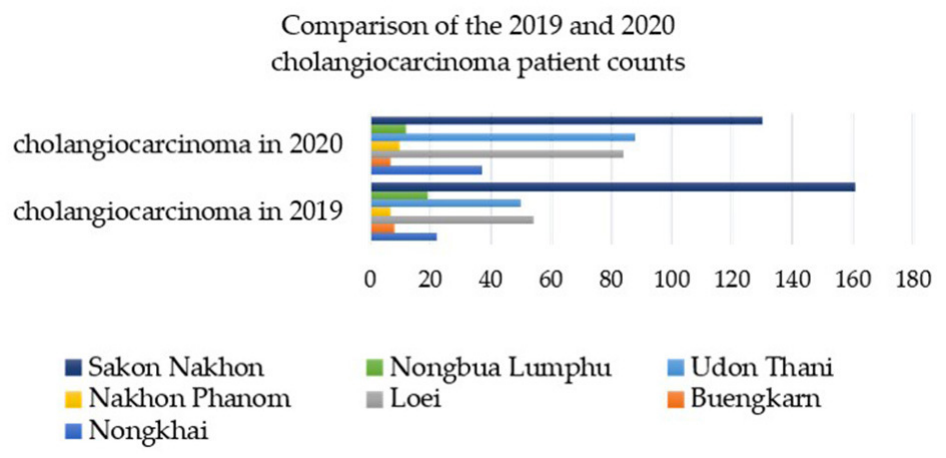
The percentage of individuals infected with liver fluke in the 8th Regional Health Province (R8) near the Mekong River between 2019 and 2020 [adatped from Pumhirunroj et al. (44)].

From 2019 to 2020, a total of 12,063 instances of stool testing were conducted at the national level and reported to the 8th Health District Office (Region 8) (40). Out of these cases, 2,832 were reported in Sakon Nakhon province, with the highest number of liver fluke infections found in the surrounding provinces within the interconnected river basin system of Nakhon Phanom and Bueng Kan (43). Figure 2 illustrates the percentage of reported cases that were discovered. Sakon Nakhon province is home to the largest freshwater resource in the northeast, providing a breeding ground for animals during the rainy season (12). Given that Phon Na Kaeo in Sakon Nakhon province has the highest average infection rate, it is crucial for provincial health officials to closely monitor the situation. Hence, data on the number of liver fluke infections in the Phon Na Kaeo district were utilized in this investigation.

### 2.3 Defining of independent variables

**Figure 3:**
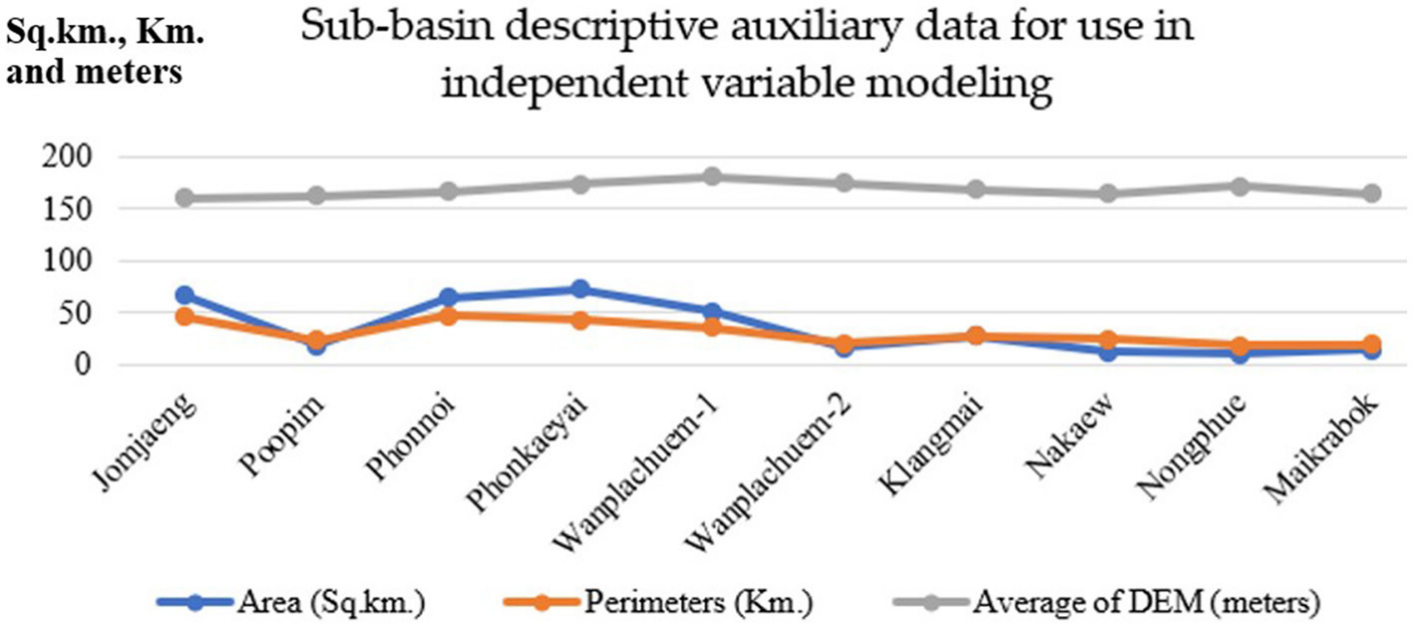
Basic data for categorizing independent variables.

The selection of independent variables is guided by prior research conducted by Pumhirunroj et al. (44), which demonstrated that using mathematical models to replicate independent variables from existing geographically information layers can improve model accuracy. Demarcating the appropriate spatial units is an important critical initial step in developing the independent variable model utilizing the clustering method. To obtain Sentinel-2 satellite imaging data from Google Earth Engine (GEE), follow these steps: (1) Define Thailand's borders using an area of interest (AOI). Set the download time period to “2019-01-01” or “2021-04-31.” (2) Filter out images with a cloud cover percentage >10%. (3) Combine the wavelengths of the Sentinel-2 image for better visualization. (4) Compute the remote sensing index utilizing surface temperature, the Normalized Difference Moisture Index (NDMI), the Normalized Difference Vegetation Index (NDVI), and the Soil-Adjusted Vegetation Index (SAVI). The data required to construct the mathematical model of independent variables includes area, perimeter, and Digital Elevation Model (DEM), utilizing slope, aspect, and curvature to represent hydrological characteristic variables, which can be extracted from the GEE system as shown in Figure 3. The area and perimeter are calculate derived from the clustering boundaries.

The rationale for selecting these nine factors, based on our area inspection, stems from our observations that the prevalence of liver flukes across all seasons was significantly associated with regions exhibiting good surface moisture retention during the dry season. We noted a positive correlation between these moisture retention areas and the number of infected individuals. Furthermore, surface moisture factors were interconnected with various land use types. The indexing of each independent variable operates at a spatial resolution of 10 m. Each factor is computed to ascertain the average distribution per sub-basin area. Additionally, Factors 6–9 were derived from remote sensing indices utilizing the raster calculator function, representing the average of Sentinel-2 imagery from January to April during the years 2019 to 2021, reflecting the dry season. This allows for an analysis of the areas where the host medium persists while awaiting the onset of the rainy season. The mathematical models have been developed from foundational factors based on extensive research concerning the variables influencing liver fluke infection in watershed areas. This is depicted in the mathematical model for calculating each factor in Equations 1–9 as follows.

The independent variable set consists of nine factors. The soil type suitable for the habitat of the intermediate host is moist clay soil, primarily found in rice field plantation areas. This indicates the suitability score for each soil type as represented by the land use types index (X1). Soil drainage factors significantly influence the suitability of the intermediate host. Specifically, soils with poor drainage can retain substantial moisture during the dry season, as indicated by the range of scores associated with the soil drainage properties index (X2). Distance from the road is another factor considered in the modeling process, as proximity between the water source and road increases the likelihood of moisture being retained in the land surface compared to greater distances. In other words, the road network influences water flow, as illustrated by the distance index from the road network (X3). The proximity of a water source is a factor that is directly linked to the increased likelihood of implantation on the surface of an intermediary host. Specifically, the closer the surface water source is, the greater the chance of implantation compared to a more distant source. This relationship is represented by the surface water source distance index (X4). The cumulative flow of water analyzed from the cumulative flow lines of the minor water lines constitutes a network designed to distribute the habitat of the intermediary host to community areas that are more distant from the main water source, with closer areas being prioritized. These flow lines are more appropriate for medium-range encounters rather than long distances, as indicated by the flow accumulation line distance index (X5). The optimal surface temperature of the host medium should not exceed 25°C at a depth of no more than 15 cm, as detected by Pumhirunroj et al. (45). By utilizing the SR index from Sentinel 2 satellite imagery, a surface temperature map can be generated. This is illustrated in the model average surface temperature index (X6). In addition to the aforementioned spatial factors, there are also remote sensing index factors such as, including the average surface moisture index (X7), the average normalized vegetation difference index (X8), and the average soil-adjusted vegetation index (X9). This study utilized 12 images from 2019 to 2021, aligning them with data on liver fluke infections in the region. The image data were then used to perform calculations. To ensure stability, all three indices were averaged to minimize fluctuations in the values. The model of independent variables is presented in detail below.


(1) $$\begin{array}{l} X_{1}=\frac{C_{i}C_{j}}{A_{k}} \end{array}$$

where *X*_1_ is a list of land use categories that are appropriate for intermediate host housing. *C*_*i*_ is any kind of land use weight value where *i* = (1 = built-up, 2 = woodland, 3 = miscellaneous, 4 = paddy field, and 5 = rice paddies in irrigated areas and water body). *C*_*j*_ is a class *j* land use area measured in square meters, and *A*_*K*_ is sub-basin area size (sq.m.) at any given *k* unit.


(2) $$\begin{array}{l} X_{2}=\frac{SO_{i}W_{j}}{A_{k}} \end{array}$$

where *X*_2_ is a measure of the drainage qualities of the soil that make it ideal for the intermediate host to live in. *SO*_*i*_ is the area of any soil type's drainage properties. *W*_*j*_ is the drainage weight value for any kind of soil. The values were weighted according to soil drainage type, indicating that well-drained soils have a lower risk of host infestation compared to poorly drained soils.


(3) $$\begin{array}{l} X_{3}=\frac{\sum_{i=1}^{n}\sum_{j=1}^{m}L_{i}B_{j}}{A_{k}} \end{array}$$

where *X*_3_ is the distance index from the road network that is used to evaluate whether the intermediary host is suitable to handle water that the road network has caught. The distance *L*_*i*_, measured in meters, is the length of the road line to any given distance *K*, which might range from 500 to more than 1,000, 1,500, 2,000, and more. The closest distance from the road, within a radius of 500 m, is the distance that receives the highest risk-weighted score, thereby establishing a standard score for proximity to the road network within the study area. At every distance k, where *K* starts at 500, 1,000, 1,500, 2,000 m, and beyond, *B*_*j*_ is the buffer distance.


(4) $$\begin{array}{l} X_{4}=\frac{\sum_{i=1}^{n}\sum_{j=1}^{m}W_{i}B_{j}}{A_{k}} \end{array}$$

When moisture still builds up during the dry season, the medium host's suitability for embedding to the soil surface is evaluated using the distance index *X*_4_ from surface water sources. *W*_*i*_ is the distance *k*, starting at 500 m, going up to 1,000, 1,500, 2,000 m, and beyond, from any surface water source *i*.


(5) $$\begin{array}{l} X_{5}=\frac{\sum_{i=1}^{n}\sum_{j=1}^{m}DW_{i}B_{j}}{A_{k}} \end{array}$$

where *X*_5_ is the distance index from the water's accumulated flow line, which is used to assess the medium host's compatibility in terms of waterlogging and moisture buildup during the dry season. *DW*_*i*_ is the distance at any distance *k* from any of the water's collected flow lines, where *k* ranges from 500 to 2,000 m and beyond.


(6) $$\begin{array}{l} X_{6}=\frac{\sum_{i=1}^{n}ST_{i}A_{ik}}{A_{k}} \end{array}$$

where *X*_6_ is the sub-basin average surface temperature index, which is used to assess if the medium host is suitable for subsurface embedding in a sub-basin. *ST*_*i*_ any surface temperature in degrees Celsius on the grid. *A*_*ik*_ is the overall temperature within the sub-basin boundary at *k*°C.

Indexes calculated from Sentinel-2 satellite images for the development of independent variables 7–9 include the NDVI, NDMI, and SAVI indices, along with the following simulation models.


(7) $$\begin{array}{l} \text{NDVI}=\left(\text{NIR}-\text{Red}\right)/\left(\text{NIR}+\text{R}\right) \end{array}$$

For Sentinel-2 the formula is: (B8 – B4)/(B8 + B4), where: B8 = 842 nm, B4 = 665 nm, NDVI range value is −1 to 1.


(8) $$\begin{array}{l} \text{NDMI}=\left(\text{NIR}-\text{SWIR}\right)/\left(\text{NIR}+\text{SWIR}\right) \end{array}$$

The wavelength range for calculating the NDMI index using Sentinel-2 images is as follows: NIR (band 8) is at 842 nm, and SWIR (band 11) is at 1,610 nm.


(9) $$\begin{array}{l} \text{SAVI}=\left(1+\text{L}\right)\;*\;\left(\text{NIR}-\text{Red}\right)/\left(\text{NIR}+\text{Red}+\text{L}\right) \end{array}$$

For Sentinel-2 the formula is: (B08 – B04)/(B08 + B04 + L) ^*^ (1.0 + L); L = 0.428, where: L is a soil brightness correction factor ranging from 0 to 1, L = 1 low vegetation cover, L = 0 high vegetation cover, L = 0.5 intermediate vegetation cover.

This includes the preparation of Sentinel-2 satellite imaging data from January to April 2019, 2020, and 2021, which coincide with the rainy and dry seasons when mollusks are buried in damp soils. A total of 12 satellite imagery data (four images per year for 3 years) were used to calculate the indices (X6) temperature index, (X7) NDMI, (X8) NDVI, and (X9) SAVI as independent variables in the GWR model. Second, screening for independent variables. And third, exploring alternative models. The detailed steps are outlined below.

(1) The normalized difference vegetation index (NDVI), which is a value that indicates the proportion of vegetation covering the surface by taking the near-infrared wave range (NIR) and the red wave range reflected from the surface to calculate the reflection difference, making the NDVI value between −1 and 1 if the plant does not have green leaves, is calculated using field surveys and GWR modeling.

Field surveys and GWR modeling were conducted to analyze the relationship between liver flukes and spatial factors. The NDVI value ranges from −1 to 1, with 0 indicating the absence of vegetation. Another index used in this research is the soil-adjusted vegetation index (SAVI), which is calculated by comparing the energy reflection in the NIR with the energy reflection in the red-light wave range, and dividing it by the total energy reflection in the NIR and the soil's energy reflection coefficient. The SAVI is obtained by multiplying the NIR by two and subtracting the square root. Both indices range from negative to maximum to 1, and the suitable index values for the habitat of the liver fluke medium host are ~-0.2 to 0.2 of the SAVI index. These values are calculated using a formula that involves doubling the NIR, squaring it, multiplying it by eight, subtracting the total red wave, and dividing it by two. The SAVI index ranges from negative to maximum to 1. It is calculated by squaring the difference between doubling the near-infrared wave (NIR) plus one and eight times the near-infrared wave (NIR), and then subtracting the total red wave divided by two. The index values that are considered suitable for the habitation of the liver fluke medium host are ~-0.2 to 0.2.


(10) $$\begin{array}{l} X_{7}=\frac{\sum_{i=1}^{n}NDMI_{i}A_{ik}}{A_{k}} \end{array}$$

where *X*_7_ is the sub-basin's average surface moisture index, which is used to assess if host media from subsurface embedding is suitable. *NDMI*_*i*_ is the value of any grid surface wetness. The entire surface moisture area at *i* that is included inside the sub-basin boundary at *k* is denoted by *A*_*ik*_.


(11) $$\begin{array}{l} X_{8}=\frac{\sum_{i=1}^{n}NDVI_{i}A_{ik}}{A_{k}} \end{array}$$

where *X*_8_ is any sub-basin's average vegetation index, which is used to assess whether the medium host is suitable for subsurface embedding in that sub-basin. *NDVI*_*i*_ can be any value for the grid-normalized differential vegetation index. Within the sub-basin boundary at *k*, *A*_*ik*_ represents the total area of the vegetation index at *i*.


(12) $$\begin{array}{l} X_{9}=\frac{\sum_{i=1}^{n}SAVI_{i}A_{ik}}{A_{k}} \end{array}$$

where *X*_9_ represents the vegetation index, which is used to modify the average soil in each sub-basin in order to assess whether the medium host from subsurface embedding in the sub-basin is appropriate. *SAVI*_*i*_ is the soil-adjusted vegetation index value for any grid. Within the sub-basin border at *k*, *A*_*ik*_ is the entire area of the soil-adjusted vegetation index at *i*.

### 2.4 GWR-based spatial modeling

**Figure 4:**
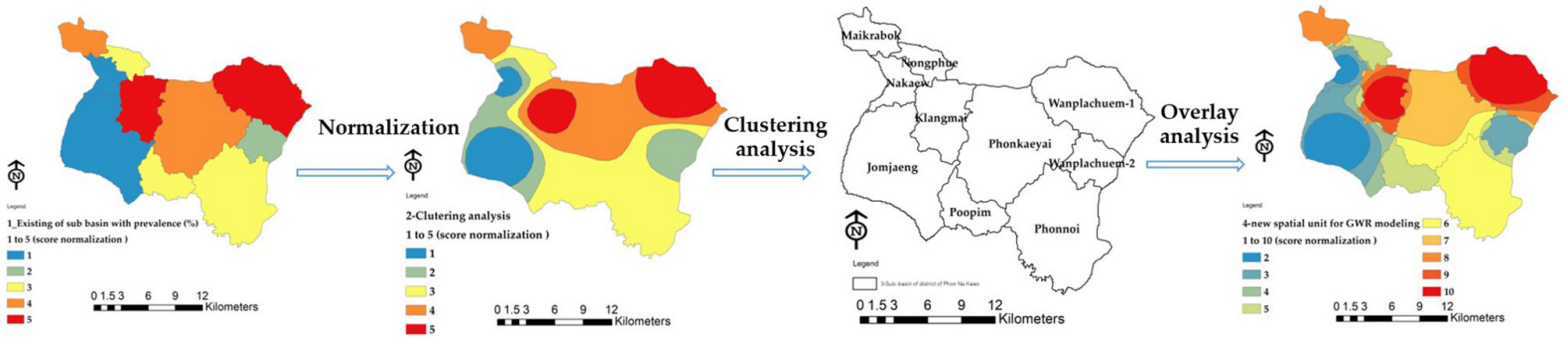
Acquiring new spatial units for GWR modeling.

Calculations of independent variables from X6 to X9 were conducted to assess surface moisture factors and surface cover indicators using satellite photos. Remote sensing data of prototype areas at the sub-basin level were analyzed through GWR modeling to investigate spatial connections to liver fluke infection (OV). The research algorithm consists of three steps: First, data collection and manipulation to study the association between liver flukes and watershed regions in sub-basins. A description of the workflow of the GWR model can be seen in Figure 4.

(2) The GWR model utilizes the same methodology as the traditional linear GWR model for coefficient estimation. However, by incorporating a geostatistical statistic, it is possible to generate a variable dataset with a smaller sample size, while still maintaining a comparable *Z*-value to the original dataset. According to Littidej and Buasri (28), the best location for shellfish implantation appears to be the buffer zone distant from the water's accumulated flow line. Variable X5 represents the independent variable group 1 (spatial variables), which is the distance index from the flow accumulation lines. The mean of the line length, or the level 3 to 3 water flow level, is a variable that indicates the likelihood of embedding the host's intermediary of liver flukes along two sides of the stream within a range of 500–2,000 m. The variable data points are generated based on the village locations where the OV data were surveyed. For each feature in the collection, GWR generates a local regression equation. When a cluster of spatial descriptive variables is available, issues with local multicollinearity are more likely to arise. The output feature class's conditional number (COND) field indicates if the outcome is unstable due to local multicollinearity.
(3) Unlike traditional models such as the global model and multiple regression method, GWR modeling uses a local model of spatial statistics. In this study, the original shape data of area units was not suitable for constructing the geographically-weighted regression (GWR) model. To address this, we developed a method for creating new area unit boundaries, allowing us to generate a set of coefficients for independent variables that align consistently with the distribution of the independent variable data layer. This means that a specific model is created for each sub-basin in order to analyze and predict the relationship between liver fluke, other parasite types, and spatial factors more accurately. GWR stands for geo-weighted regression model. In contrast to the original method (OLS), where a model is obtained to predict every unit area with different coefficients, In the study, GWR employs the distance reciprocal weighting method to establish the coefficient of the relationship between the independent and dependent variables (29, 30, 46). Based on this research, it is recommended that GWR modeling incorporates a data layer that considers additional spatial factors, such as proximity to roads and water bodies, as well as the prevalence of liver fluke infection in the sub-basin region. This analysis can be conducted using 5-m DEM data, and the independent variables can be created using mathematical functions that correlate satellite image wavelengths. Equation 10 provides a visual representation of how the GWR model estimates the regression coefficient for each survey point or linear regression point. This estimation is achieved by analyzing a polylinear regression equation and applying sub-spatial statistics to determine the relationship between the independent and dependent variables (35) (Equation 13).
(13) $$\begin{array}{l} Y_{i}=\beta_{0}\left(u_{i}v_{i}\right)+\beta_{1}\left(u_{i}v_{i}\right)x_{1}+\beta_{2}\left(u_{i}v_{i}\right)x_{2}....+\beta_{k}\left(u_{i}v_{i}\right)x_{k}\\ \;+\varepsilon_{i} \end{array}$$

where (*u*_*i*_*v*_*i*_) are the orthogonal coordinates at each linear regression point. β_*k*_(*u*_*i*_*v*_*i*_) is the regression coefficient estimated at each linear regression point. At each linear regression point, the regression coefficient (β) of each independent variable (*X*) is estimated as a matrix of *n*×*(k* +*1)*.

The GWR model is an analysis of multiple linear regression equations at each linear regression point that must be weighted to focus on the data (29, 47–50). The regression coefficient is then estimated, as shown in Equation 14.


(14) $$\begin{array}{l} \beta_{\left(i\right)}={\left(X^{T}W\left(i\right)X\right)}^{-1}X^{T}W\left(i\right)y \end{array}$$

In this study, three approaches were utilized, as illustrated in Equations 13–16. We used Equations 15, 16 to calculate the root mean square error (RMSE) and coefficient of determination (*R*^2^).


(15) $$\begin{array}{l} RMSE=\sqrt{\frac{\sum_{i=1}^{m}{\left(\rho_{i}-\sigma_{i}\right)}^{2}}{m}} \end{array}$$

Where, ρ_*i*_ = prediction, σ_*i*_ = existing value, m = total count of data.


(16) $$\begin{array}{l} R^{2}=1-\frac{\sum_{i=1}^{m}{\left(\rho_{i}-\sigma_{i}\right)}^{2}}{\sum_{i=1}^{m}{\left(z-\sigma_{i}\right)}^{2}} \end{array}$$

In order to assess the accuracy of the GWR, we used the Receiver Operating Characteristic-Area Under Curve (ROC-AUC) technique. This method is commonly employed in machine learning to evaluate the precision of various models and identify any issues related to interpretation and criteria selection (51). ROC curves are created by plotting the True Positive Rate (TPR), also known as sensitivity, against the False Positive Rate (FPR), which represents specificity. The TPR, located on the y-axis, measures the proportion of existing positives accurately detected, while the FPR, on the x-axis, measures the proportion of negative instances or non-events incorrectly classified as positive or events (52). Moreover, the ROC curve also indicates how frequently the model incorrectly predicts a positive outcome when the true outcome is negative (53). The Equations 17, 18 can be used to calculate the TPR and FPR.


(17) $$\begin{array}{l} TPR=\frac{TP}{TP+FN} \end{array}$$

(18) $$\begin{array}{l} FPR=\frac{FP}{FP+FN} \end{array}$$

TP represents the count of correctly predicted positive instances, FN represents the count of actual positive instances that were incorrectly predicted as negative, FP represents the count of actual negative instances that were incorrectly predicted as positive, and TN represents the count of correctly predicted negative instances. After calculating TPR (True Positive Rate) and FPR (False Positive Rate), an AUC (Area Under the Curve) value of 50% indicates that the estimation lacks discrimination (53). However, if the AUC exceeds 90%, the model can be considered to have exceptional effectiveness (54). The positional data analyzed by AUC is derived from the predictions generated by the GWR model, expressed as a percentage of infections. A value of (+/-0.5%) is designated as the infection threshold for a given point when it is not classified as infected, and this is compared with the actual percentage of infected sites. It is important to note that the impact of these independent variables on dependent variables may vary across different sub-regions (spatial units). Consequently, agencies or organizations can utilize these analysis results to effectively manage parasite infection prevention systems, provided they are able to assess the spatial characteristics of parasite species distribution (55). This proactive approach enables communities to preserve their wellbeing and reduce healthcare expenses by preventing future illnesses.

### 2.5 An optimized spatial unit for GWR modeling

The original sub-basin boundary only covers an area of 10 units, which is insufficient for building the GWR model. To address this, a guideline is proposed to increase the number of area units. This can be achieved by using the attribute values of the nine independent variables to adjust the standard value within the range of 0–1. The risk of infection in the water source is analyzed, and the standardized value is used for further analysis. The Clustering Getis-Ord Gi^*^ method is then employed to demonstrate the grouping of independent variables that influence liver leafworm infection in water bodies. The resulting groups are superimposed on the existing 10 boundaries to obtain a new boundary for creating the GWR model. This leads to an increase in the area units from the original 10 units to 33 units. Refer to Figure 4 for the sub-operation diagram illustrating this process.

## 3 Results

### 3.1 Distribution of variables

**Figure 5:**
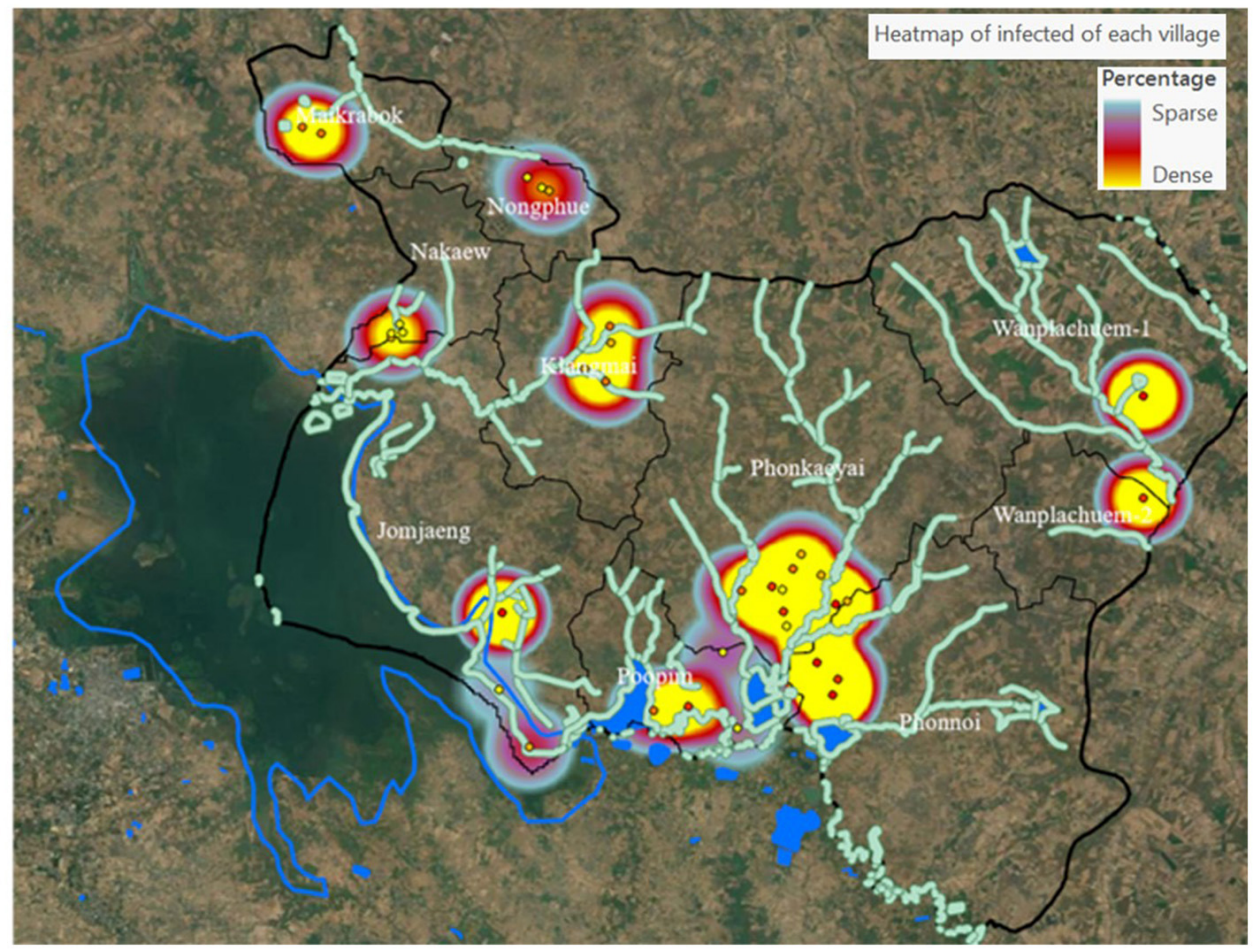
The heat map of prevalence of ov-infection in term of percentage Y(prevalence of OV, %).

The variables used in the GWR model are obtained by generating raster data using a heat map approach, as depicted in Figure 5. The average value of the raster represents the percentage likelihood of water source infection in the 10 subbasins. These values will then be converted to match the boundaries of the 33 new area units, resulting in the variable values. The heat map's raster data needs to have a cell size of 10 m to match the grid size of the Sentinel-2. The corresponding variable represents the total number of grids obtained from this heatmap, and it is calculated as an average for the boundaries of each subbasin reconstructed using the spatial boundary design approach.

**Figure 6:**
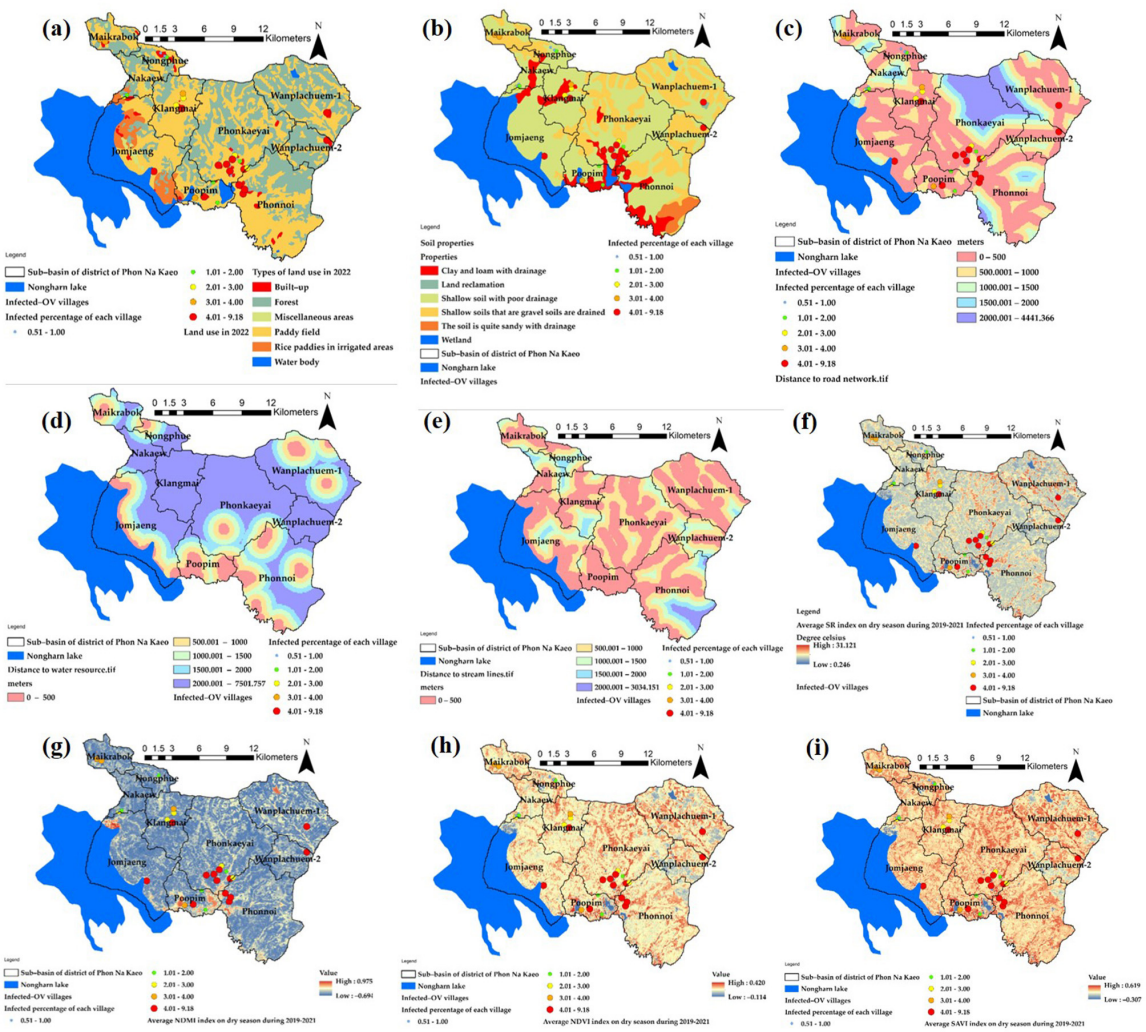
Derived index map using mathematical model of *X*_1_–*X*_9_ **(a–i)**.

Figure 6 displays the descriptive data values for the index values of the nine independent variables that were used to construct mathematical models from Equations 1–9. Preparing independent variable sets using ArcGIS Pro version 3.2.0 was a crucial phase in the GIS process. This phase involved utilizing various techniques of spatial data interpolation to generate multi-raster and vector datasets. The percentage of cases in Wanplachuem-1, Phonnoi, and Wanplachuem-2 sub-basins was extremely high, with values of 9.18, 7.84, and 6.489, respectively. These three watersheds are close to each other and joined by an outlet. When considering the values of nearly all the indices, Wanplachuem-2 is more valuable than other river basins. This is mainly because the index's value is divided by the smaller basin area more than in the case of the other basins. For Jomjaeng, Phonnoi, and Phonkaeyai, the spatial units of the sub-basin with comparable island index values of the X1 index are 14.773, 17.688, and 14.279, respectively.

The index values of X2 for Wanplachuem-2, Klangmai, and Nakaew are 24.128, 24.577, and 29.858, respectively. The island groups Jomjaeng, Phonnoi, Phonkaeyai, and Wanplachuem-1, as well as X3, X4, and X5, are all located in the same basin. Although the groups of remote-sensing indices are not significantly different from each other, they still need to be studied along with other variables in GWR modeling. Another round of correlation analysis is also necessary to check for duplicate factors. It is important to use mathematical models to standardize data across different groups of factor index values. Standardizing the data to a comparable range improves the accuracy of building and fitting models in GWR. Instead of directly importing raw data into models, this approach yields better results.

The primary distribution of the intermediate index on the map is shown by the X6 index. Figure 6f shows yellow regions with flat surfaces between 26 and 28°C, while red regions with high temperatures primarily consist of constructions like roads and villages. The X7 index depicts the distribution of high-level indices, which are areas near water bodies with index values >0.6 or more, and indicate ideal habitat substrate host areas. The X8 and X9 indices, both composed of vegetation index, have similar distributions. However, the X9 index adds a constant value to the vegetation value to increase reflectivity, and both can be used interchangeably. Correlation findings from consistency can be seen to validate the modeling, and the red areas in both indices indicate suitable locations that resemble the X7 index.

**Figure 7:**
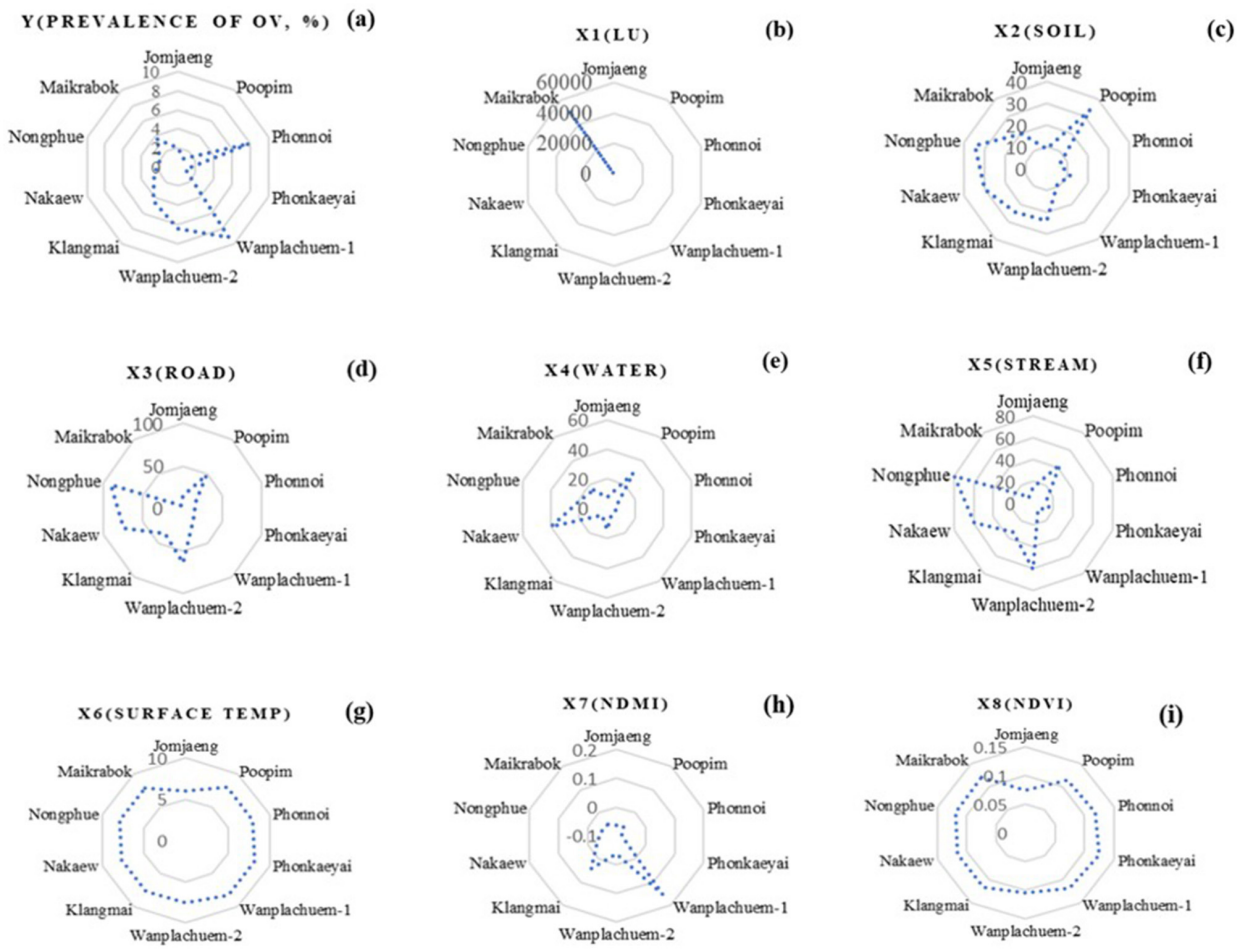
Derived index radar graph using mathematical model of *X*_1_–*X*_9_ **(a–i)**.

The raster map data results of the X1 variant were dispersed across most sub-basins and within a buffer distance of up to 500 m. These results were similar to the X3 index values, except for the upper basin areas where the index values were low due to the absence of road networks. In the lower sections, which are near sizable freshwater marshes, the X4 and X5 index map values indicated high scores mainly in the lower basin and scattered low values in the higher parts. Figure 7 presents the results in both a map and radar graph format. The map illustrates the distribution of the independent variables, while the graph demonstrates how this distribution varies across different sub-basin boundaries. This visual representation helps to highlight the extent of the map effect.

### 3.2 Selected the influence factors associated with spatial liver fluke (Opisthorchis viverrini) infection

**Figure 8:**
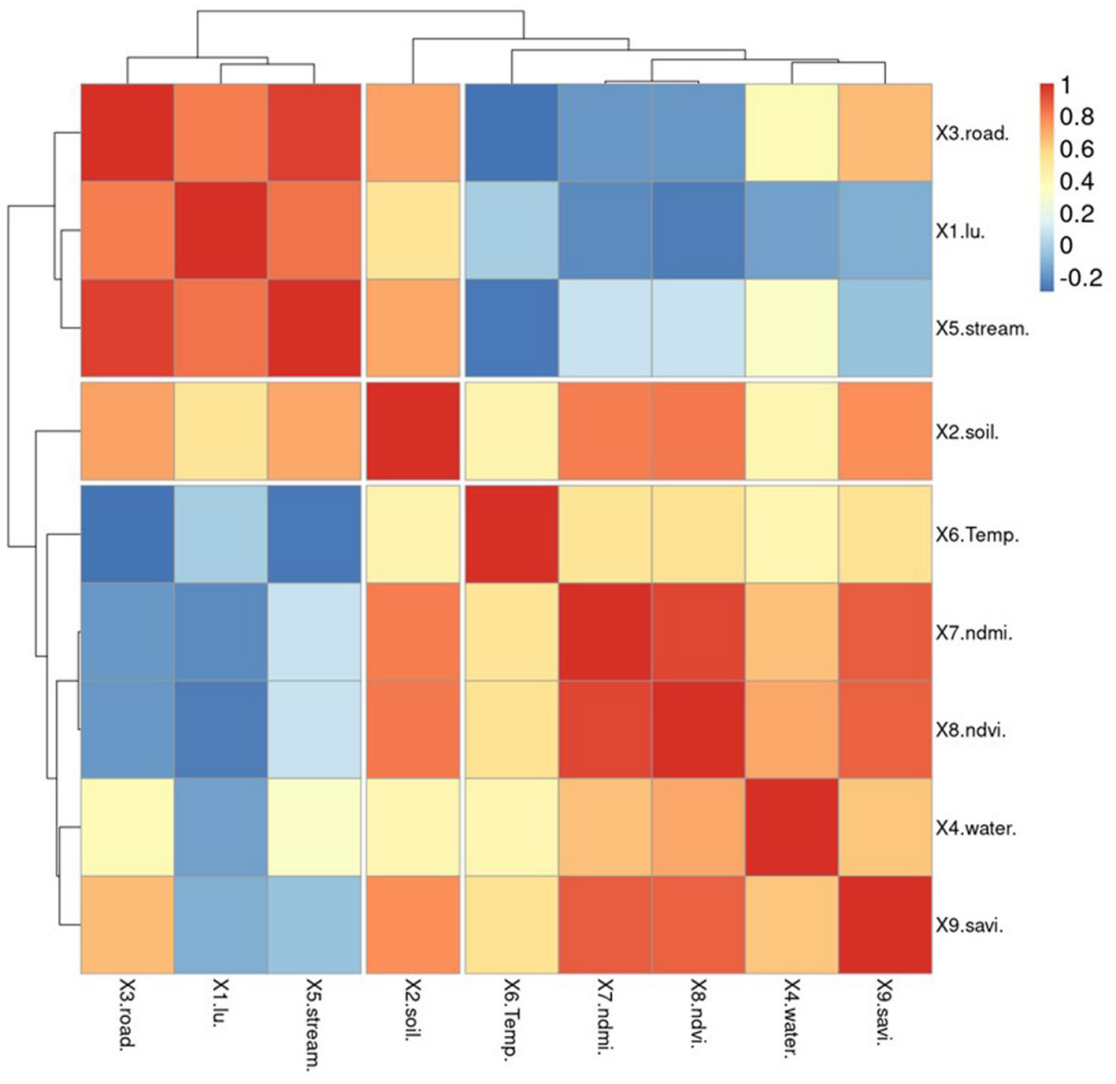
The correlation matrix of independent variables.

To ensure that GWR models can maintain acceptable *R*^2^ values, it is important to reduce redundancy in the number of independent variables. In this study, spatial correlation analysis was used to screen for independent variables. The independent variable set is divided into two categories: variables generated from vector data (represented by points, polylines, and polygons) and solving factors derived from vector data (represented by X1–X5). This type of data can be imported and examined alongside other variables without the need to create raster data or assign score values based on quantifiable requirements in advance. X6–X9 are already raster data, but they were mathematically created to normalize the data and enable correlation with the preceding set of variables. Figure 8 demonstrates that the percentage of individuals infected with OV is inversely related to factors X5 and X9. The analysis of variable groupings using a dendrogram reveals three main groups based on their correlations. The first group consists of variables that have a high correlation with each other, specifically variables X2 and X9. The second group includes independent variables X5, X7, and X9, while the third group consists of independent variables X5, X7, and X8. Notably, variable X9 is a key factor in the association with infections identified in past tests of Pumhirunroj et al. (44, 45). These groupings are represented as Model-1, Model-2, and Model-3, respectively.

This means that the risk of infection increases as one moves further away from this set of characteristics, but the likelihood of contracting the fluke decreases. Factors X1 and X2 indicate that the risk of infection increases with poor drainage, as poorly drained soil retains more moisture. Additionally, the use of agricultural land near irrigation canals leads to increased moisture on the soil surface compared to other types of land. Factor X5, which correlates with the percentage of infected individuals (0.226), can be used as a representation of factors X1–X4 in the analysis of vector factors, where X1, X2, X3, and X4 have correlation values of 0.985, 0.838, 0.984, and 0.612, respectively.

Apart from being screened, the independent variables X5–X9 were also used to generate correlation graphs for examining the regression of the GWR model. Two techniques were employed to identify reliability patterns through residual plot graph analysis. The first technique involved plotting the residuals, which are estimates of the difference between the observed values of Y (% of OV) and the fitted values. These residuals should be dispersed randomly when observations occur. The second technique involved plotting the normal probability plots of the errors and the expected values. If the plot resembles a straight line, it indicates that the disparities have a normal distribution. As mentioned in the section, the X5 variable set shows a normal distribution of the data, as illustrated in Figure 8. This table demonstrates how the vertical distribution of information for variables X6–X9 translates into a limited range of index values that can predict the proportion of infections across a wide range.

### 3.3 The best GWR model for liver fluke (Opisthorchis viverrini) infection prediction

Comparing multiple alternative models increases the likelihood of selecting the correct model for prediction (56, 57). In spatial factor correlation simulation, an independent set of variables is used as an alternative to GWR modeling. This allows for the visualization of tolerance patterns at the small area unit level. Correlation analysis was conducted to select the independent variables to be integrated into GWR models. Specifically, variables X5–X9 were chosen, simulated, and presented in Table 1. Based on the analysis results, a suitable GWR model with a high *R*^2^ value was identified for forecasting the percentage of infected individuals. This variable demonstrates a high level of significance, as indicated by the extremely high t-statistics or extremely low *p*-values (58, 59). The results presented in the table compare the precision between GWR and OLS (ordinary least square) models, offering a visual representation of the differences in accuracy between the two models (60).

**Table 1:** Results of GWR for selected alternative model.

**GWR-models**	**Factors**	**Coefficients**	***t*-stat**	***p*-value**	***R*^2^ (OLS)**	**Avg-*R*^2^ (GWR)**	**RMSE (%)**	**AUC**
Model-1	Intercept	15.632	4.634^***^	0.000^***^	0.511	0.535	3.442	0.512
		X2(soil)	−1.414	−0.905 n/s	0.234 n/s				
		X9(savi)	−6.019	−2.031 n/s	0.125 n/s				
Model-2	Intercept	60.236	3.029^***^	0.000^***^	0.762	0.817	2.465	0.675
		X5(stream)	−5.036	−2.071^***^	0.046^***^				
		X7(ndmi)	4.287	1.794^***^	0.036^***^				
		X9(savi)	−9.795	−2.698 n/s	0.186 n/s				
Model-3	Intercept	62.467	1.851^***^	0.000^***^	0.754	0.800	2.571	0.659
		X5(stream)	−5.167	−2.045^***^	0.016^***^				
		X7(ndmi)	1.118	0.784^***^	0.085^***^				
		X8(ndvi)	−3.109	−0.851 n/s	1.072 n/s				

^***^Significant at 5% level.

n/s, not significant.

Results of Monte Carlo test for spatial non-stationarity.

Model-1, Model-2, and Model-3. To assess the negative projection of the findings from GWR Model-1 as a percentage of infected individuals, two independent variables, X2 and X9, were imported. The spatial non-stationarity test table presents results from Monte Carlo (22, 28), and compares *R*^2^ values to those of OLS models. The model exhibits negative coefficients of −1.414 and −6.019 on the respective scales, accompanied by *t*-stat values of −0.905 and −2.031, and *p*-values of 0.234 and 0.125. These results suggest that there is currently no significant (n/s) correlation between the two parameters and the proportion of infected individuals. In addition, the model reveals an *R*^2^ value for the GWR model, which stands at a higher level of 0.535 compared to the OLS model's 0.511. Since both components exhibit a respectable level of relationship with *R*^2^, further examination is needed for the second alternative model.

The second GWR model (Model-2) shows a negative and positive correlation between the components X5 and X7, while the X9 factor reveals a negative relationship. This suggests that the percentage of infected people decreases as the area of separation between vegetation covers increases. The X9 factor is statistically significant with a *p*-value of 0.186, indicating that the likelihood of individuals with liver fluke infection increases with mid-range and less-than-peak soil correction index factors. The t-statistic for X9 is also larger than the other two factors (−2.698). The accuracy of the model, as measured by *R*^2^, improves to 0.762 and 0.817. Although factors X5 and X8 have been accepted and tested for t-stat and *p*-value, both of which have a negative impact on infection, it is essential to test these two factors in Model-3 to confirm their suitability as alternative predictors for sub basin infection. In alternative Models-3 when the X8 component is included. Both the X5 and X8 coefficients exhibit a negative trend and have more significant *t*-statistics and *p*-values compared to the other variables. Model-3 is considered the best GWR model for predicting case percentage because it maintains a confidence level >80% without including too many independent variables that could lead to inaccurate predictions. Although Model-2 has a higher *R*^2^ value, it may introduce duplication of the independent variable set and result in coincidental relationships, which could inflate the *R*^2^ value.

**Figure 9:**
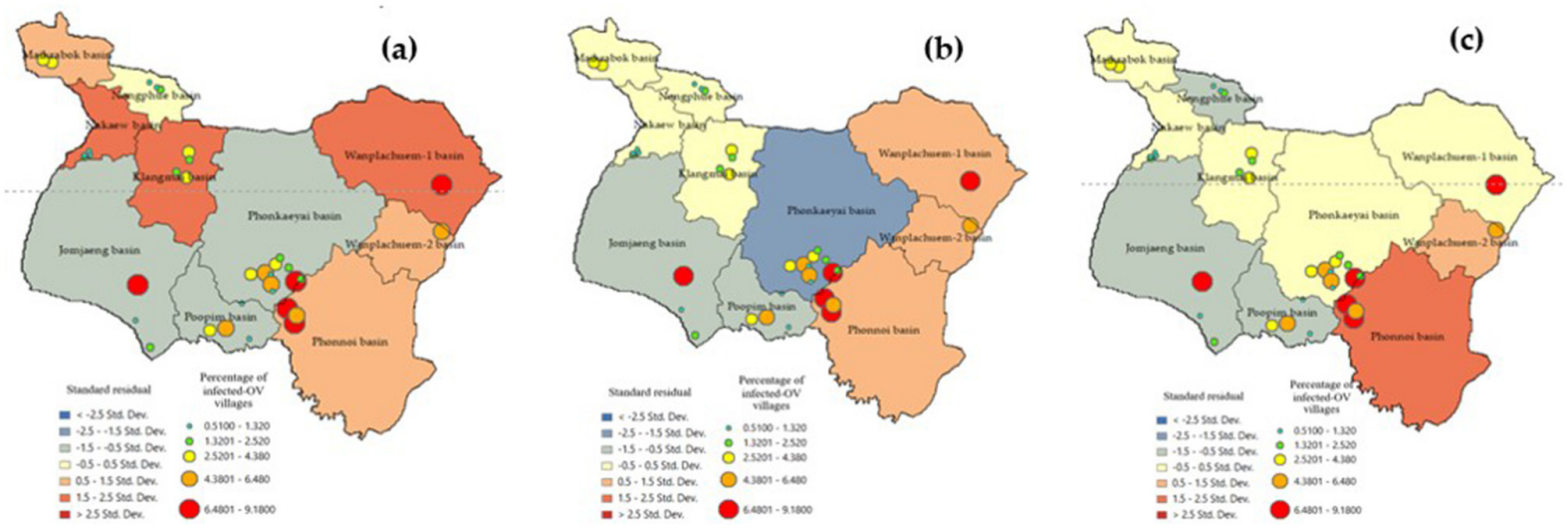
Comparison of standard residual of alternative GWR-model: **(a)** Model-1, **(b)** Model-2, and **(c)** Model-3.

The standard residual index (SR) is a commonly used measure to assess the prediction accuracy of a model. It provides an index value that indicates the accuracy of the model, with values displayed in intervals of 0.5 (28, 35), Figure 9 shows the depiction of these values. Sub-basin units with SR values between −0.5 and 0.5 are considered areas where GWR models can make accurate predictions with smaller tolerances compared to other locations. In the case of the GWR Model-3, the sub-basins Maikrabok, Klangmai, Nongphue, Phonkaeyai, and Wanplachuem-1 are highlighted in yellow. These sub-basins have a tolerance that is five units lower than the other models. Additionally, the SR results from GWR Model-3 further support the idea that the deviation area has the same direction and can help minimize the discrepancy in sub-basin areas. Furthermore, the results obtained from the SR analysis using the Model-2 model continue to support the conclusion that the Maikrabok, Klangmai, and Nongphue river basins are comparatively lower than other river basins and display less variation. These findings reinforce the suitability of the Model-3 model when it comes to lower tolerances compared to the two alternative models. The results of this analysis using the SR index were used to develop a policy aimed at reducing the suitability of embedding the medium host in moist soils.

**Figure 10:**
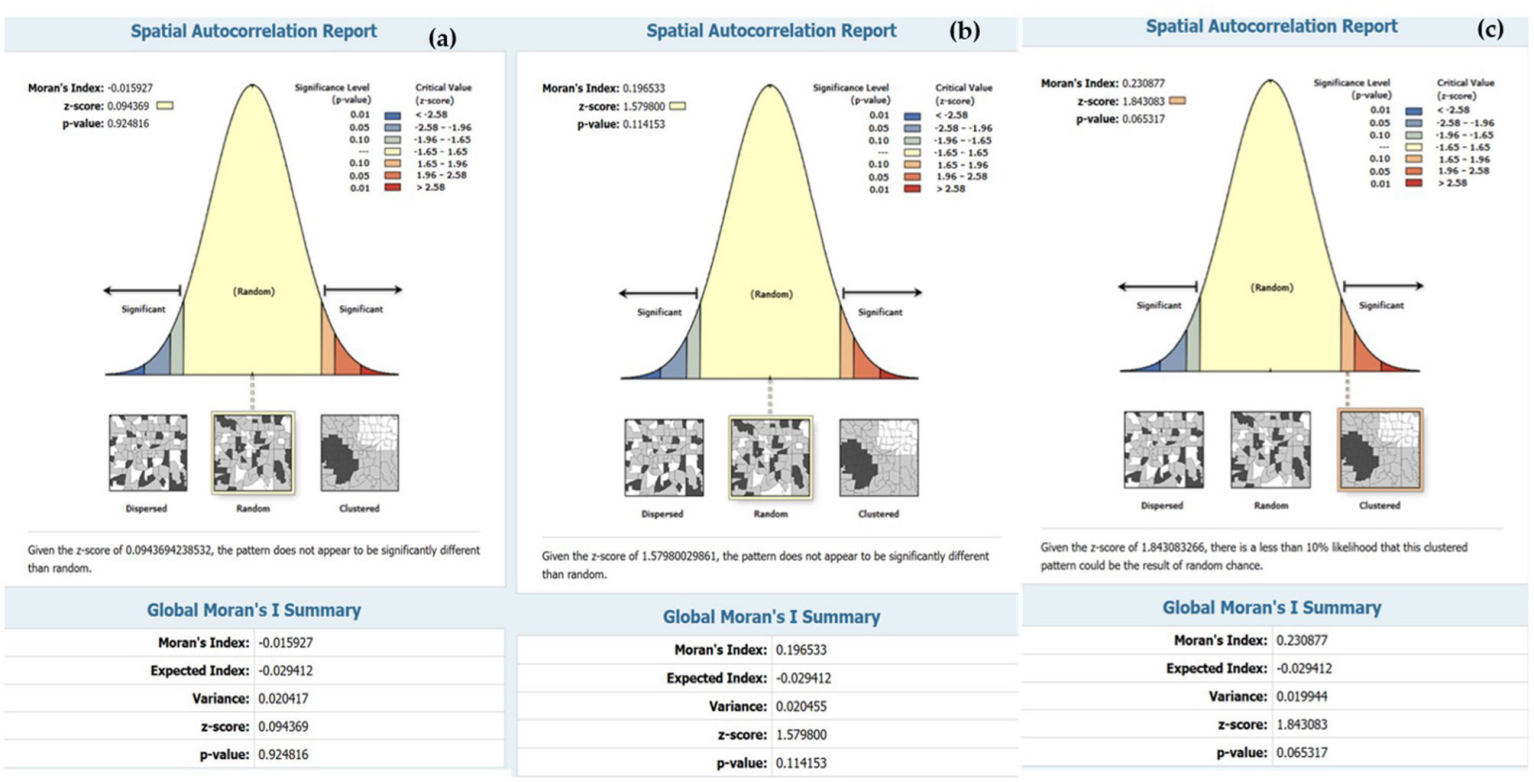
Result of spatial autocorrelation index **(a)** predicted by Model-1, **(b)** predicted by Model-2, and **(c)** predicted by Model-3.

The accuracy of the prediction results was compared to the existing infection levels to determine if sub-basin locations with poor tolerances could show a spatial correlation map in an island group. The SR map of the forecast results using Model-3. The figures illustrate that various factor, like densely distributed water flow lines, affect the moisture retention of surface water during droughts. Moreover, road networks that intersect with water flow channels play a significant role in elevating the risk of infection in this region (45). The Phonkhaeyai basin exhibits a narrow range of SR, indicating the reliability of the prediction results, unlike the forecasts for other watersheds that show similarities. Consequently, it can be inferred that the upper and middle basins, situated farther away from Nongharn, might experience elevated rates of liver fluke infection. This is due to the independent variables that transport water and fish to the vicinity of the road fold area, which acts as an ideal moisture reservoir. To ensure the reasonable predictability of Model-3, it is important to check the coherence of infection percentages in the prediction results. This can be done by comparing the percentage of current infections. The prediction results from various models and SR data are combined to create a spatial correlation index. The results show that the geographically correlation between the observed OV infection percentages, predicted percentages, and SR is −0.015927, 0.196553, and 0.230877, respectively. The study not only analyzed the residual values but also indicated the likelihood of differences in predictions across each area unit, which does not guarantee the consistency of these predictions. To tackle this issue, a spatial autocorrelation analysis was performed. While the AUC values of Model-3 and other models were comparable, a comparison with Moran's I index showed only slight differences in AUC. Additionally, Model-3 displayed a cluster of residual values, reinforcing the model's effectiveness by suggesting that these area units yielded values close to the actual infection rates. The distribution patterns are illustrated in Figure 9 and they appear to be random, random, and clustered, depicted in Figure 10.

**Figure 11:**
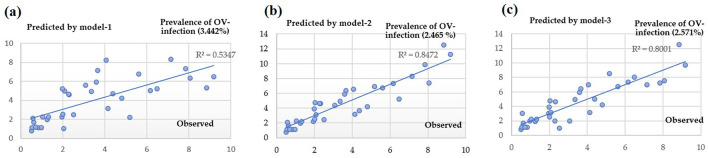
Comparison of RMSE of **(a)** Model-1, **(b)** Model-2, and **(c)** Model-3.

**Figure 12:**
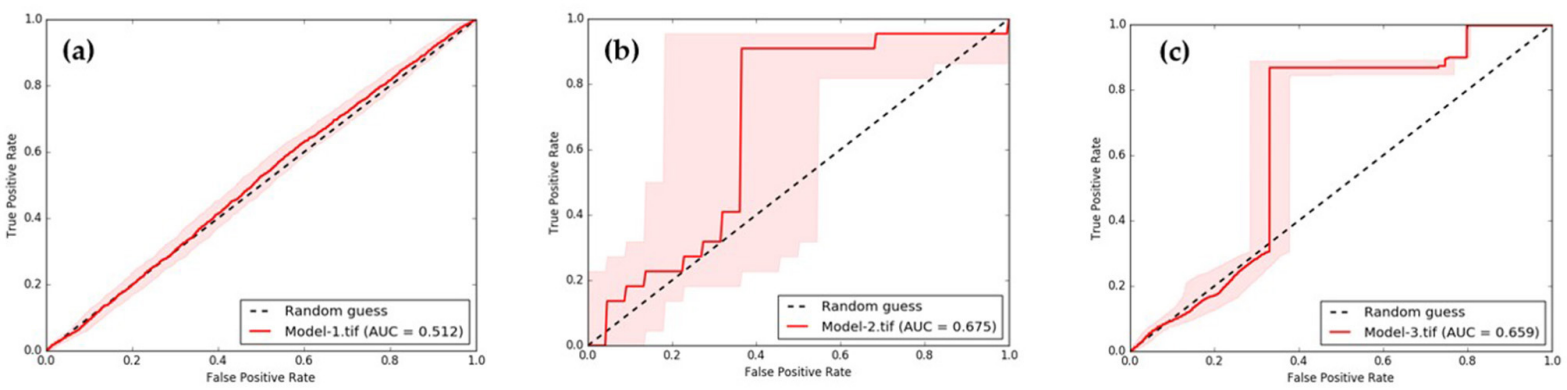
Comparison of AUC of **(a)** predicted by Model-1, **(b)** predicted by Model-2, and **(c)** predicted by Model-3.

The results in Figure 11 indicate that Model-1, Model-2, and Model-3 have RMSE (root mean square error) values of 3.442, 2.465, and 2.571%, respectively. The corresponding *R*^2^ values are 0.5347, 0.8472, and 0.8001. To evaluate the overall model accuracy. Based on the RMSE test results, it is evident that both Model-2 and Model-3 yield similar results. Consequently, it becomes challenging to determine which model performs better, leading to the need for an additional test method using AUC (area under curve). The AUC value ranges from 0 to 1, with 1 representing the highest performance in classification, 0.5 indicating random classification, and values below 0.5 suggesting a less efficient model. Figure 12 demonstrates this classification efficiency. Since the infection percentage variable is not initially represented as binary values (infection vs. non-infection), it needs to be adjusted and converted into a binary dataset for comparison with the actual infection data. However, the approach to model development can identify a set of independent variables that consistently correlate with infection predictions.

**Figure 13:**
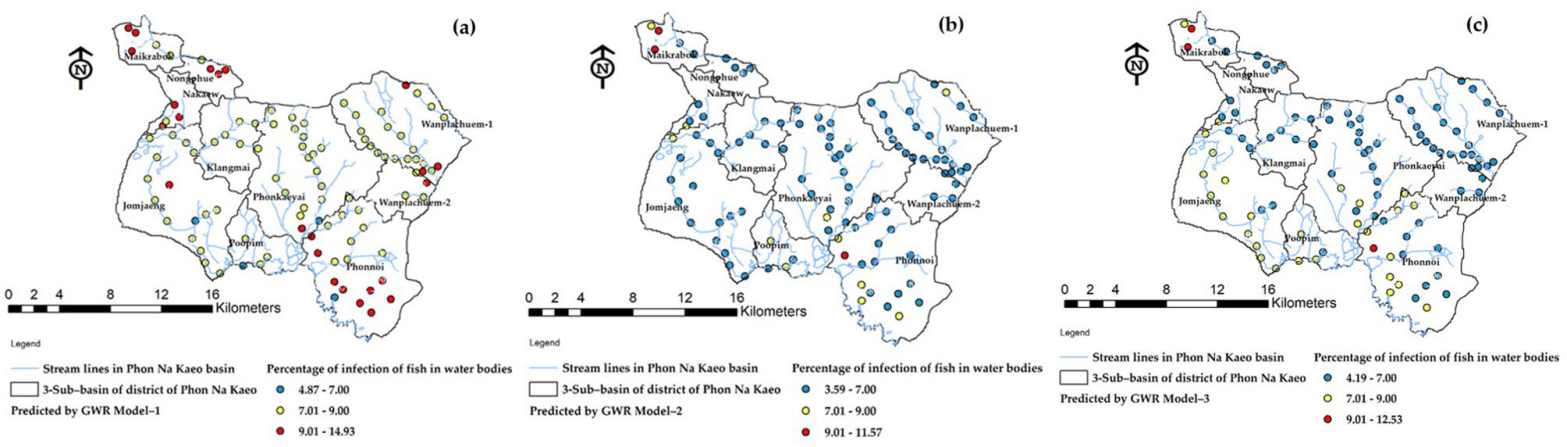
The mapping of ov-infected prediction **(a)** predicted by Model-1, **(b)** predicted by Model-2, and **(c)** predicted by Model-3.

The AUC results of the three models indicate that Model-2, Model-3, and Model-1 performed the best, with performance values of 0.675, 0.659, and 0.512, respectively. Furthermore, upon comparing the predicted range in the red bar shown in Figure 12, it is evident that the deviation range of Model-2 is larger than that of almost every data range used for testing. Therefore, it can be concluded that the Model-3 is the most suitable for predicting ov-infections, as illustrated in Figure 13.

The forecast results indicate the location of the river's flow line within each sub-basin, which serves to connect the water flow to the community area. The positioning of infection simulation points is determined based on the accessibility of fishing locations for the public and the link between the stream and the river bank. Figure 13 reveals that the Model-3 model, which has been deemed the most effective, predicts a risk value for the water source ranging from 7.01 to 9.00%. This range of risk levels is crucial to monitor as it represents the largest distribution of locations and covers the most area. However, it is still important to consider the potentially risky effects from the other two models for observation. Additionally, other test results such as SR, spatial correlation, RMSE, etc., should be taken into account.

## 4 Discussion

### 4.1 Independent variable set redundancy

X3, X4, and X5 are vector-type independent variables that are automatically connected geographicallyally. These variables are redundant because the method used to analyze the data calculates the distance from the vector data, generates a score range based on the distance of infection risk, and uses the Euclidean distance function. Before adding the three independent variables to the model, only the representative factor, X5, needs to be chosen. This factor must be distinct from the X1 and X2 datasets, which determine scoring values differently based on their relationship to infection. There are additional duplicated variables in the raster variable set created from satellite imagery indices, including X6, X8, and X9. The X7 component can also contribute to the model, but it does not improve accuracy when included. It is automatically correlated when observed by correlation. Therefore, using the independent variables X5, X7, and X9 yields the best modeling results. Despite being less than the bulk inputs in Model 4, the results of *R*^2^, *t*-stat, and *p*-value statistics were sufficient to validate the choice of models and a suitable range of independent variables for forecasting liver fluke cases in small basin systems. To create GWR models, which divide unit areas into sections based on the distribution of dependent variables and produce precise results, it is crucial to mathematically model independent variable data to meet quantifiable standards.

### 4.2 Design of spatial units

In order to specify the amount of data, it is necessary to draw boundaries for spatial subspace units. The analysis results were obtained from 10 sub-basin boundaries, which were distributed based on the flow sequence level (3–6) from upstream to downstream at the marshes shown in Figure 4. Other descriptive information about the sub-basin, such as its size, was also included. In this study, digital elevation model (DEM) data with a cell size of 12.5 m was utilized to generate the sub-basin layer data. Perimeter length and average height of the unit area are presented in Table 2. The fill and sink tool were used to readjust the spatial height of the DEM dataset, which is a technique for hydrological analysis that realistically analyzes the altitude data using GIS procedures. This allows for continuous analysis of water flow. The Wanplachuem-1 river basin had the highest average elevation (180.397 m), followed by the Wanplachuem-2 and Phonkaeyai basins. These basins had elevations of 174.412 and 172.894 m, respectively. The upper basin of the Phon Na Kaeo district is also at this higher elevation. However, despite being in the upper basin, a significant portion of the population in these areas is still affected by OV infection. The upper basin is prone to flooding during different seasons, allowing carp groups and intermediate host mollusks to migrate to these areas for feeding. The Wanplachuem-1 river basin has the highest average height (180.397 m), followed by the Wanplachuem-2 and Phonkaeyai basins with measurements of 174.412 and 172.894 m, respectively. The upper basin of the Phon Na Kaeo district is also considered to be at this height. Despite being in the upper basin, a significant portion of the population in these areas is infected with OV. The upper basin is prone to floods during different seasons, which attracts carp groups and intermediate host mollusks for feeding. The average calculation for all sub-basins is consistent due to the continuous number of infected individuals displayed in the case percentage data. However, the average may vary depending on the values used to compute the raster. The *Z*-value represents the percentage of affected individuals in the hamlet. To incorporate all details in the raster data, a radius of 4 km is used to generate a heatmap for the raster map. Yellow areas indicate high density and a higher probability of coming into contact with infected individuals, while green areas show low percentages of infected individuals (Figure 5). In order to analyze positional data and other raster data of independent variables and create trend graphs, the GWR model requires a continuous value of raster data, which can be achieved by creating heatmaps of infected individuals.

**Table 2:** Sub-basin descriptive auxiliary data for use in independent variable modeling.

**Sub-basin name**	**Area (sq.km.)**	**Perimeters (km.)**	**Average of DEM (m.)**
Jomjaeng	66.183	46.434	160.445
Poopim	18.432	23.986	161.624
Phonnoi	64.081	46.779	166.147
Phonkaeyai	72.794	43.279	172.894
Wanplachuem-1	51.264	36.087	180.397
Wanplachuem-2	16.071	20.354	174.412
Klangmai	27.738	28.053	167.640
Nakaew	12.708	24.316	164.155
Nongphue	10.04	18.698	170.894
Maikrabok	14.259	19.312	163.884

### 4.3 Model capabilities and other areas' development approaches

The GWR model utilizes the Gaussian model to enhance the data by increasing the number of cells and graphing the trend of independent variables more effectively compared to other models. This is achieved through the calculation of boundary distance from the location of an infected person, resulting in the generation of raster data. The changes in data are then analyzed to identify trends (56, 57). The optimization method used by the GWR model has a benefit of ensuring the continuation of the data surface. Additionally, it screens independent variables that strongly connect fluke infection with *t*-stat and *p*-value indices, reducing duplication and keeping the model compact. Creating geographicallyal units from an independent set of variables' actual correlation is crucial when using the GWR model to forecast the percentage of fluke infections in a local area. The Sakon Nakhon Provincial Public Health Office, an organization in the area with extensive knowledge on liver fluke infection, approved the input of independent variables in this study. However, the organization was interested in understanding the intricate relationships between spatial variables to formulate policies and conduct spatial analysis to lower the number of infected individuals.

### 4.4 Guidelines for using the model to inform provincial public health policy

The guidelines for preventing and controlling liver fluke and bile duct cancer, as provided by the Sakon Nakhon Provincial Public Health Office (40), include various measures. These measures encompass the implementation of sanitation systems and sewage management to disrupt the parasite cycle, health literacy education in schools, liver fluke screening for individuals over 15 years old, bile duct cancer screening for individuals aged 40 and above who are at risk and have undergone ultrasounds, systematic management of referrals for suspected cholangiocarcinoma for diagnosis and treatment, safe food practices, and a campaign aimed at eradicating parasites from fish. Furthermore, the guidelines include a system for receiving and referring patients from hospitals to communities, and reporting their performance through the Ministry of Public Health's reporting system or the Isan Cohort database (21). According to a study utilizing a spatial model, this approach can support sanitation and sewage management strategies in breaking the parasite cycle (12). Moreover, the GWR model, which tracks the number of infected individuals, can be employed to examine trends by continually collecting data.

## 5 Conclusion

This study created a geographicallyally weighted regression (GWR) model to monitor liver fluke infection. The results were compared to determine the accuracy and suitability of the spatial statistical model compared to ordinary least squares (OLS) models in similar studies. The findings indicated that the GWR model is more accurate and appropriate for investigating liver fluke infection at the local level (28, 29, 31). To fully utilize the model, it is essential to first construct a spatial unit data layer that appropriately and independently separates the variables (49, 50, 61). In many cases, GWR models yield low coefficients of determination due to improper subarea unit allocations. However, this study proposes establishing sub-basin units with continuous nearby boundaries as a potential strategy to study spatial correlations with liver fluke infections. Therefore, this work can serve as a prototype for such investigations. In order to establish a relationship between the percentage of infected individuals and a set of independent factors, it is important to regularly collect local fluke case data. Future predictions should incorporate factors that are consistent with infection in both stream variables. Additionally, SAVI should develop forecasts using alternative models, such as machine learning techniques, including logistic models that generate probabilistic dependent variable datasets, thereby enhancing the realism of the forecasts. In more advanced research, it would be beneficial to use spatial survey parameters, such as soil wetness in the field where mollusks are located, instead of the factors used in this study, which are only prototypes for testing the GWR model. Another approach to improving the model's prediction is to use mathematical modeling to adjust the measurements in the database so that they can be analyzed together (30). Ultimately, the findings of this research can guide the development of spatial models on a small-watershed scale to monitor liver fluke infections in other regions with similar watershed characteristics.

## Data Availability

The original contributions presented in the study are included in the article/Supplementary material, further inquiries can be directed to the corresponding author.

## References

[1] Saengsawang P Promthet S Bradshaw P. Infection with Opisthorchis viverrini and use of praziquantel among a working-age population in Northeast Thailand. Asian Pacific J Cancer Prev. (2013) 14:2963–6. 10.7314/APJCP.2013.14.5.2963 23803062

[2] Saengsawang P Promthet S Bradshaw P. Prevalence of OV infection in Yasothon Province, Northeast Thailand. Asian Pacific J Cancer Prev. (2012) 13:3399–402. 10.7314/APJCP.2012.13.7.3399 22994767

[3] Chai JY Han ET Guk SM Shin EH Sohn WM Yong TS. High prevalence of liver and intestinal fluke infections among residents of Savannakhet Province in Laos. Kor J Parasitol. (2007) 45:213–8. 10.3347/kjp.2007.45.3.213 17876167 PMC2526321

[4] Sithithaworn P Haswell-Elkins M. Epidemiology of Opisthorchis viverrini. Acta Trop. (2003) 88:187–94. 10.1016/j.actatropica.2003.02.001 14611873

[5] Sripa B Bethony JM Sithithaworn P Kaewkes S Mairiang E Loukas A. Opisthorchiasis and opisthorchis-associated cholangiocarcinoma in Thailand and Laos. Acta Tropica. (2011) 120(Suppl.):S158–68. 10.1016/j.actatropica.2010.07.006 20655862 PMC3010517

[6] Songserm N Promthet S Sithithaworn P Pientong C Ekalaksananan T Chopjitt P. Risk factors for cholangiocarcinoma in high-risk area of Thailand: role of lifestyle, diet and methylenetetrahydrofolate reductase polymorphisms. Cancer Epidemiol. (2012) 36:e89–94. 10.1016/j.canep.2011.11.007 22189445

[7] Vennervald BJ Polman K. Helminths and malignancy. Parasite Immunol. (2009) 31:686–96. 10.1111/j.1365-3024.2009.01163.x 19825108

[8] Wongratanacheewin S Sermswan RW Sirisinha S. Immunology and molecular biology of Opisthorchis viverrini infection. Acta Trop. (2003) 88:195–207. 10.1016/j.actatropica.2003.02.002 14611874

[9] Smout MJ Sripa B Laha T Mulvenna J Gasser RB Young ND. Infection with the carcinogenic human liver fluke, Opisthorchis viverrini. Mol Biosyst. (2011) 7:1367–75. 10.1039/c0mb00295j 21311794 PMC3739706

[10] Poomphakwaen K Promthet S Kamsa-Ard S Vatanasapt P Chaveepojnkamjorn W Klaewkla J. Risk factors for cholangiocarcinoma in Khon Kaen, Thailand: a nested case-control study. Asian Pacific J Cancer Prev. (2009) 10:251–8. 19537893

[11] Sripa B Pairojkul C. Cholangiocarcinoma: lessons from Thailand. Curr Opin Gastroenterol. (2008) 24:349–56. 10.1097/MOG.0b013e3282fbf9b3 18408464 PMC4130346

[12] Perakanya P Ungcharoen R Worrabannakorn S Ongarj P Artchayasawat A Boonmars T. Prevalence and risk factors of Opisthorchis viverrini infection in Sakon Nakhon Province, Thailand. Trop Med Infect Dis. (2022) 7:6–8. 10.3390/tropicalmed7100313 36288054 PMC9607628

[13] Sadaow L Rodpai R Janwan P Boonroumkaew P Sanpool O Thanchomnang T. An innovative test for the rapid detection of specific IgG antibodies in human whole-blood for the diagnosis of Opisthorchis viverrini infection. Trop Med Infect Dis. (2022) 7:308. 10.3390/tropicalmed7100308 36288049 PMC9607866

[14] Boonjaraspinyo S Boonmars T Ekobol N Artchayasawat A Sriraj P Aukkanimart R. Prevalence and associated risk factors of intestinal parasitic infections: a population-based study in Phra Lap Sub-District, Mueang Khon Kaen District, Khon Kaen Province, Northeastern Thailand. Trop Med Infect Dis. (2023) 8:10022. 10.3390/tropicalmed8010022 36668929 PMC9860576

[15] Prasongwatana J Laummaunwai P Boonmars T Pinlaor S. Viable metacercariae of Opisthorchis viverrini in Northeastern Thai cyprinid fish dishes–as part of a rational program for control of O. viverrini-associated cholangiocarcinoma. Parasitol Res. (2013) 112:1323–7. 10.1007/s00436-012-3154-9 23052784

[16] Sripa B Kaewkes S Sithithaworn P Mairiang E Laha T Smout M. Liver fluke induces cholangiocarcinoma. PLoS Med. (2007) 4:e201. 10.1371/journal.pmed.0040201 17622191 PMC1913093

[17] Sripa B Brindley PJ Mulvenna J Laha T Smout MJ Mairiang E. The tumorigenic liver fluke *Opisthorchis viverrini* multiple pathways to cancer. Trends Parasitol. (2012) 28:395–407. 10.1016/j.pt.2012.07.006 22947297 PMC3682777

[18] Haswell-Elkins MR Satarug S Elkins DB. Opisthorchis viverrini infection in Northeast Thailand and its relationship to cholangiocarcinoma. J Gastroenterol Hepatol. (1992) 7:538–48. 10.1111/j.1440-1746.1992.tb01035.x 1327263

[19] Mairiang E Elkins DB Mairiang P Chaiyakum J Chamadol N Loapaiboon V. Relationship between intensity of Opisthorchis viverrini infection and hepatobiliary disease detected by ultrasonography. J Gastroenterol Hepatol. (1992) 7:17–21. 10.1111/j.1440-1746.1992.tb00928.x 1311966

[20] Pumhirunroj B Aukkanimart R. Liver fluke-infected cyprinoid fish in Northeastern Thailand (2016–2017). Southeast Asian J Trop Med Public Health. (2017) 51:1–7. Available at: https://journal.seameotropmednetwork.org/index.php/jtropmed/issue/view/6

[21] Pinlaor S Onsurathum S Boonmars T Pinlaor P Hongsrichan N Chaidee A. Distribution and abundance of Opisthorchis viverrini metacercariae in cyprinid fish in Northeastern Thailand. Korean J Parasitol. (2013) 51:703–10. 10.3347/kjp.2013.51.6.703 24516277 PMC3916461

[22] Suwannatrai AT Thinkhamrop K Clements ACA Kelly M Suwannatrai K Thinkhamrop B. Bayesian spatial analysis of cholangiocarcinoma in Northeast Thailand. Sci Rep. (2019) 9:1–10. 10.1038/s41598-019-50476-7 31582774 PMC6776517

[23] Hasegawa S Ikai I Fujii H Hatano E Shimahara Y. Surgical resection of hilar cholangiocarcinoma: analysis of survival and postoperative complications. World J Surg. (2007) 31:1258–65. 10.1007/s00268-007-9001-y 17453285

[24] Geadkaew-Krenc A Krenc D Thanongsaksrikul J Grams R Phadungsil W Glab-ampai K. Production and immunological characterization of ScFv specific to epitope of Opisthorchis viverrini rhophilin-associated tail protein 1-like (OvROPN1L). Trop Med Infect Dis. (2023) 8:160. 10.3390/tropicalmed8030160 36977161 PMC10055880

[25] Thinkhamrop K Suwannatrai AT Chamadol N Khuntikeo N Thinkhamrop B Sarakarn P. Spatial analysis of hepatobiliary abnormalities in a population at high-risk of cholangiocarcinoma in Thailand. Sci Rep. (2020) 10:16855. 10.1038/s41598-020-73771-0 33033306 PMC7545164

[26] Pratumchart K Suwannatrai K Sereewong C Thinkhamrop K Chaiyos J Boonmars T. Ecological niche model based on maximum entropy for mapping distribution of bithynia siamensis goniomphalos, first intermediate host snail of Opisthorchis viverrini in Thailand. Acta Trop. (2019) 193:183–91. 10.1016/j.actatropica.2019.03.004 30849302

[27] Martviset P Phadungsil W Na-Bangchang K Sungkhabut W Panupornpong T Prathaphan P. Current prevalence and geographic distribution of Helminth infections in the parasitic endemic areas of rural Northeastern Thailand. BMC Publ Health. (2023) 23:448. 10.1186/s12889-023-15378-4 36882723 PMC9993603

[28] Littidej P Buasri N. Built-up growth impacts on digital elevation model and flood risk susceptibility prediction in Muaeng District, Nakhon Ratchasima (Thailand). Water. (2019) 11:71496. 10.3390/w11071496

[29] Littidej P Uttha T Pumhirunroj B. Spatial predictive modeling of the burning of sugarcane plots in Northeast Thailand with selection of factor sets using a GWR model and machine learning based on an ANN-CA. Symmetry. (2022) 14:101989. 10.3390/sym14101989

[30] Prasertsri N Littidej P. Spatial environmental modeling for wildfire progression accelerating extent analysis using geo-informatics. Pol J Environ Stud. (2020) 29:3249–61. 10.15244/pjoes/115175

[31] Sangpradid S. Application of a multi-layer perceptron neural network to simulate spatial-temporal land use and land cover change analysis based on cellular automata in Buriram Province, Thailand. Environ Eng Manag J. (2023) 22:917–31. 10.30638/eemj.2023.074

[32] Littidej P Kromkratoke W Pumhirunroj B Buasri N Prasertsri N Sangpradid S. Enhanced rubber yield prediction in high-density plantation areas using a GIS and machine learning-based forest classification and regression model. Forests. (2024) 15:1535. 10.3390/f15091535

[33] Lu B Charlton M Fotheringham AS. Geographically weighted regression using a non-euclidean distance metric with a study on London House Price Data. Proc Environ Sci. (2011) 7:92–7. 10.1016/j.proenv.2011.07.017

[34] Lu B Charlton M Harris P Fotheringham AS. Geographically weighted regression with a non-euclidean distance metric: a case study using hedonic house price data. Int J Geograph Inform Sci. (2014) 28:660–81. 10.1080/13658816.2013.865739

[35] Fotheringham A Charlton M. Geographically Geographically Weighted Weighted Regression Regression A Stewart Fotheringham (2014).

[36] Suwannahitatorn P Webster J Riley S Mungthin M Donnelly CA. Uncooked fish consumption among those at risk of Opisthorchis viverrini infection in Central Thailand. PLoS ONE. (2019) 14:e0211540. 10.1371/journal.pone.0211540 30703149 PMC6355008

[37] Sripa B Kaewkes S Intapan PM Maleewong W Brindley PJ. Chapter 11—Food-borne trematodiases in Southeast Asia: epidemiology, pathology, clinical manifestation and control. In:Zhou XN Bergquist R Olveda R Utzinger JBTA, editors. Important Helminth Infections in Southeast Asia: Diversity and Potential for Control and Elimination, Part A. Cambridge, MA: Academic Press (2010). p. 305–50. 10.1016/S0065-308X(10)72011-X 20624536

[38] Qian MB Utzinger J Keiser J Zhou XN. Clonorchiasis. Lancet. (2016) 387:800–10. 10.1016/S0140-6736(15)60313-0 26299184

[39] Brindley PJ Bachini M Ilyas SI Khan SA Loukas A Sirica AE. Cholangiocarcinoma. Nat Rev Dis Prim. (2021) 7:2. 10.1038/s41572-021-00300-2 34504109 PMC9246479

[40] Pumhirunroj B Littidej P Boonmars T Bootyothee K Artchayasawat A Khamphilung P. Machine-learning-based forest classification and regression (FCR) for spatial prediction of liver fluke *Opisthorchis viverrini* (OV) infection in small sub-watersheds. ISPRS Int J GeoInform. (2023) 12:503. 10.3390/ijgi12120503

[41] Dao TTH Bui TV Abatih EN Gabriël S Nguyen TTG Huynh QH. Opisthorchis viverrini infections and associated risk factors in a lowland area of Binh Dinh Province, Central Vietnam. Acta Tropica. (2016) 157:151–7. 10.1016/j.actatropica.2016.01.029 26872984

[42] Ruantip S Eamudomkarn C Kopolrat KY Sithithaworn J Laha T Sithithaworn P. Analysis of daily variation for 3 and for 30 days of parasite-specific IgG in urine for diagnosis of strongyloidiasis by enzyme-linked immunosorbent assay. Acta Trop. (2021) 218:105896. 10.1016/j.actatropica.2021.105896 33753029

[43] Honjo S Srivatanakul P Sriplung H Kikukawa H Hanai S Uchida K. Genetic and environmental determinants of risk for cholangiocarcinoma via Opisthorchis viverrini in a densely infested area in Nakhon Phanom, Northeast Thailand. Int J Cancer. (2005) 117:854–60. 10.1002/ijc.21146 15957169

[44] Pumhirunroj B Littidej P Boonmars T Bootyothee K Artchayasawat A Khamphilung P. Machine-learning-based forest classification and regression (FCR) for spatial prediction of liver fluke Opisthorchis viverrini (OV) infection in small sub-watersheds. ISPRS Int J Geo-Informat. (2023) 12:v1. 10.20944/preprints202308.2039.v1

[45] Pumhirunroj B Littidej P Boonmars T Artchayasawat A Prasertsri N Khamphilung P. Spatial predictive modeling of liver fluke Opisthorchis viverrine (OV) infection under the mathematical models in hexagonal symmetrical shapes using machine learning-based forest classification regression. Symmetry. (2024) 16:81067. 10.3390/sym16081067

[46] Sripa B Tangkawattana S Laha T Kaewkes S Mallory FF Smith JF. Toward integrated opisthorchiasis control in Northeast Thailand: the Lawa project. Acta Trop. (2015) 141:361–7. 10.1016/j.actatropica.2014.07.017 25102053 PMC4454771

[47] Lord J Odoi A. Determinants of disparities of diabetes-related hospitalization rates in Florida: a retrospective ecological study using a multiscale geographically weighted regression approach. Int J Health Geogr. (2024) 23:1. 10.1186/s12942-023-00360-5 38184599 PMC10771651

[48] Oshan TM Smith JP Fotheringham AS. Targeting the spatial context of obesity determinants via multiscale geographically weighted regression. Int J Health Geogr. (2020) 19:11. 10.1186/s12942-020-00204-6 32248807 PMC7132879

[49] Leong YY Yue JC. A modification to geographically weighted regression. Int J Health Geograph. (2017) 9:1–18. 10.1186/s12942-017-0085-9 28359282 PMC5439144

[50] Isazade V Qasimi AB Dong P Kaplan G Isazade E. Integration of Moran's I, geographically weighted regression (GWR), and ordinary least square (OLS) models in spatiotemporal modeling of COVID-19 outbreak in Qom and Mazandaran Provinces, Iran. Model Earth Syst Environ. (2023) 2023:1729. 10.1007/s40808-023-01729-y 36820101 PMC9930702

[51] Gorsevski PV Brown MK Panter K Onasch CM Simic A Snyder J. Landslide detection and susceptibility mapping using LiDAR and an artificial neural network approach: a case study in the Cuyahoga Valley National Park, Ohio. Landslides. (2016) 13:467. 10.1007/s10346-015-0587-0

[52] Fan J Upadhye S Worster A. Understanding receiver operating characteristic (ROC) curves. Can J Emerg Med. (2006) 8:19–20. 10.1017/S1481803500013336 17175625

[53] Choubin B Borji M Mosavi A Sajedi-Hosseini F Singh VP Shamshirband S. Snow avalanche hazard prediction using machine learning methods. J Hydrol. (2019) 577:123929. 10.1016/j.jhydrol.2019.12392934225139

[54] Fawcett T. An introduction to ROC analysis. Pattern Recognit Lett. (2006) 27:861–74. 10.1016/j.patrec.2005.10.010

[55] Zhao TT Feng YJ Doanh PN Sayasone S Khieu V Nithikathkul C. Model-based spatial-temporal mapping of opisthorchiasis in endemic countries of Southeast Asia. eLife. (2021) 10:e59755. 10.7554/eLife.59755 33432926 PMC7870142

[56] Brunton LA Alexander N Wint W Ashton A Broughan JM. Using geographically weighted regression to explore the spatially heterogeneous spread of *Bovine tuberculosis* in England and Wales. Stochast Environ Res Risk Assess. (2017) 31:339–52. 10.1007/s00477-016-1320-9

[57] Rujirakul R Ueng-arporn N Kaewpitoon S Loyd RJ Kaewthani S Kaewpitoon N. GIS-based spatial statistical analysis of risk areas for liver flukes in Surin Province of Thailand. Asian Pac J Cancer Prev. (2015) 16:2323–6. 10.7314/APJCP.2015.16.6.2323 25824758

[58] Brunsdon C Fotheringham S Charlton M. Geographically weighted regression-modelling spatial non-stationarity. J Royal Stat Soc Ser D. (1998) 47:431–43. 10.1111/1467-9884.00145

[59] Comber A Brunsdon C Charlton M Dong G Harris R Lu B. A route map for successful applications of geographically weighted regression. Geograph Anal. (2023) 155–78. 10.1111/gean.12316

[60] Lu B Hu Y Murakami D Brunsdon C Comber A Charlton M. High-performance solutions of geographically weighted regression in R. Geo-spat Inform Sci. (2022) 25:536–49. 10.1080/10095020.2022.2064244

[61] Düzgün HS Kemeç S. Spatial and geographically weighted regression BT—encyclopedia of GIS. In:Shekhar S Xiong H, editors. Boston, MA: Springer US (2008). p. 1073–7.

